# Comparative analysis of primary and secondary metabolites in wheat seedlings ( *Triticum aestivum* L.) cultivated under varying photosynthetic photon flux densities and growth periods

**DOI:** 10.1002/jsfa.14432

**Published:** 2025-06-13

**Authors:** Ye Jin Kim, Yu‐Mi Shin, HanGyeol Lee, So‐Yeon Moon, Tae Jin Kim, Sang Un Park, Woo Duck Seo, Jae Kwang Kim

**Affiliations:** ^1^ Division of Life Sciences Incheon National University Incheon Republic of Korea; ^2^ Division of Crop Foundation National Institute of Crop Science, Rural Development Administration Wanju Republic of Korea; ^3^ Using Technology Development Department, Bio‐resources Research Division Nakdonggang National Institute of Biological Resources Sangju Republic of Korea; ^4^ Department of Crop Science Chungnam National University Daejeon Republic of Korea; ^5^ Convergence Research Center for Insect Vectors, College of Life Sciences and Bioengineering Incheon National University Incheon Republic of Korea

**Keywords:** wheat seedling, photosynthetic photon flux density, growth period, metabolic profiling, primary metabolite, secondary metabolite

## Abstract

**BACKGROUND:**

To elucidate the impact of environmental factors on the nutritional quality of metabolites in wheat seedlings, both primary and secondary metabolites must be comprehensively examined. We thus conducted a comparative analysis of these metabolites in wheat seedlings grown under various photosynthetic photon flux densities (PPFD) (200, 400 and 800 μmol m^−2^ s^−1^) and growth periods (5, 7, 9, 11, 13 and 15 days) to help optimize the nutritional quality of seedlings.

**RESULTS:**

In total, 74 metabolites were characterized, including 51 primary metabolites and 23 secondary metabolites. Multivariate analyses confirmed changes in flavonoids, which are known antioxidants, under the various PPFD conditions. Wheat seedlings grown at a PPFD of 400 μmol m^−2^ s^−1^ for more than 9 days exhibited a higher flavonoid content than those grown under the other conditions. Furthermore, an increase in cysteine and methionine metabolism, sugar metabolism, and carotenoid and phenylpropanoid biosynthesis was observed.

**CONCLUSION:**

A PPFD of 400 μmol m^−2^ s^−1^ increases sugar metabolism in wheat seedlings by promoting anabolism through photosynthesis. Furthermore, the accumulation of sugars, the primary raw materials for secondary metabolites, affects flavonoid biosynthesis. These results suggest that wheat seedlings undergo metabolic reprogramming in response to light intensity, enabling adaptive responses. Our study thus confirmed the relationships between primary and secondary metabolites in wheat seedlings under various PPFD conditions. Furthermore, the insights gained enhance our understanding of the influence of light intensity and duration on metabolite accumulation, which is vital for optimizing agricultural practices. © 2025 The Author(s). *Journal of the Science of Food and Agriculture* published by John Wiley & Sons Ltd on behalf of Society of Chemical Industry.

## INTRODUCTION

Wheat seedlings have been attracting attention as a health food as a result of their high levels of vitamins, macronutrients, enzymes and amino acids.^1^, ^2^ The cultivation of wheat seedlings allows for precise control of various growth parameters such as water saturation, temperature, light, humidity and growth period.^3^ Light intensity and quality markedly affect plant metabolism, including photosynthesis, sugar production, and biosynthesis and accumulation of secondary metabolites.^4^, ^5^ Therefore, understanding the effects of photosynthetic photon flux density (PPFD) is vital for optimizing plant growth conditions. PPFD refers to the number of photons reaching 1 m^2^ area per second, and the measurement of this parameter allows for precise control of light intensity.^4^, ^5^, ^6^ Wheat seedlings require specific light intensity levels at different growth stages to maximize their photosynthesis and development.^7^ In wheat, light intensity selectively affects the distribution and redistribution of macro‐ and micronutrients^8^ and, in sprouts and microgreens, the accumulation of health‐promoting secondary metabolites differs between PPFD conditions.^9^ Wheat seedlings exhibit antioxidant properties, which substantially increase during germination, primarily attributed to the formation of flavonoids, a type of secondary metabolites.^10^ Thus, metabolic changes in wheat seedlings in response to PPFD need to be elucidated to help optimize the production of health‐promoting ingredients; however, this aspect remains largely unexplored.

Metabolomics serves as a useful tool to detect plant response to environmental changes and elucidate the underlying biological mechanisms.^11^, ^12^, ^13^ Furthermore, a comprehensive understanding of the biosynthetic pathways linking primary and secondary metabolites is necessary, considering that primary metabolites are the precursors of secondary metabolites.^14^ In Arabidopsis, different light intensities resulted in differences in sugar, tricarboxylic acid (TCA) cycle intermediates, and fatty acid contents^15^; sugars were more abundant in Arabidopsis grown under high light conditions (500–600 μmol m^−2^ s^−1^), whereas alanine, glutamine, glutamic acid and aspartic acid were more abundant in those grown under normal growth light conditions (130 μmol m^−2^ s^−1^).^15^ High light intensity generally promotes the production of secondary metabolites, whereas, in some plants such as sciophytes, including ginseng, the production of secondary metabolites is promoted under low light intensity. Thus, the optimal light conditions for plant growth and development differ among taxa.^16^

Our previous study confirmed that differences in phenolic compounds in wheat seedlings were more pronounced between growth periods than between cultivars.^17^ This suggests that environmental factors play a crucial role in determining the nutritional composition of wheat seedlings. However, only a few studies have explored complex metabolic networks through primary and secondary metabolite profiling in wheat seedlings. The present study was thus conducted to investigate the nutritional quality of wheat seedlings through a comprehensive analysis of the changes in primary and secondary metabolites in response to different levels of PPFD and growth periods. To this end, we cultivated wheat seedlings using different PPFD conditions (200, 400 and 800 μmol m^−2^ s^−1^) and growth periods (5, 7, 9, 11, 13 and 15 days). Subsequently, primary and secondary metabolite profiling based on gas chromatography (GC) and liquid chromatography‐mass spectrometry (MS) was performed. Changes in the metabolites of wheat seedlings according to environmental factors were investigated using multivariate analysis. The results of this study are expected to significantly improve the general understanding of plant cultivation parameters for optimizing production.

## MATERIALS AND METHODS

### Chemicals and reagents

Sigma‐Aldrich (St Louis, MO, USA) supplied ribitol, methoxyamine chloride, ascorbic acid, 6‐methoxyflavone, pyridine, fatty acid methyl ester (FAME) mixture (C8–C24), 5α‐cholestane, pentadecanoic acid and *N*‐methyl‐*N*‐trimethylsilyl trifluoroacetamide (MSTFA). Methanol and ethanol were obtained from Daejung (Kyungki‐do, Republic of Korea), whereas chloroform was sourced from Burdick and Jackson (Muskegon, MI, USA). Thermo Fisher Scientific (Waltham, MA, USA) supplied hydrochloric acid, toluene and hexane, and potassium hydroxide (KOH) was obtained from Wako (Wako Pure Chemical Corporation, Osaka, Japan). Thermo Fisher Scientific also provided methoxyamine hydrochloride (MOX), and *trans*‐*β*‐apo‐8‐carotenal was obtained from CaroteNature (Münsingen, Switzerland). Unless otherwise noted, all compounds used in this study were HPLC grade.

### Sample preparation

The Korean wheat cultivar Saekeumkang was used in this research. It was planted in 2023 with artificial soil under controlled growth chamber conditions. The growth conditions for all plants were maintained at a relative humidity of 60–70%, temperature of 18–22 °C, and a short‐day photocycle of 9 h of light and 15 h of darkness. Plants were subjected to different PPFD conditions, comprising 200, 400 and 800 μmol m^−2^ s^−1^, based on the typical light intensity range observed under field cultivation conditions (PPFD 150–900 μmol m^−2^ s^−1^).^7^, ^8^ These conditions were chosen to represent low to high light intensities. The wheat seedlings were harvested at six distinct growth stages (5, 7, 9, 11, 13 and 15 days post germination) and were initially air‐dried under natural light and temperature for 3 days at 25 °C. Thereafter, the samples were freeze‐dried at −78 °C and stored at −80 °C until analysis.

### Extraction and analysis of primary metabolites

Analyses of hydrophilic substances (free amino acids, sugars, sugar alcohols, and organic acids) were performed as described previously.^18^ Briefly, 10 mg of ground freeze‐dried wheat seedling samples was combined with 1 mL of a methanol:water:chloroform (2.5:1:1, v:v:v) solution in a 2‐mL tube. As an internal standard (IS), 0.06 mL of ribitol (0.2 mg mL^−1^ in methanol) was added. After drying, the concentrated samples were derivatized with 0.08 mL of MOX (20 mg mL^−1^ in pyridine) and then treated with 0.08 mL MSTFA. Hydrophilic metabolites were examined using a GC‐time‐of‐flight MS Benchtop (LECO, St Joseph, MI, USA) and a model 7890B GC (Agilent, Santa Clara, CA, USA) fitted with a CP‐SIL 8 CB column (CP5860; Agilent); 1 μL of derivatized sample was injected, and split mode was used at a 1:25 ratio. The temperatures of the injection port, transfer line and ion source were set to 230, 250 and 250 °C, respectively. The oven temperature was maintained at 80 °C for 2 min, then increased at a rate of 15 °C min^−1^ to 320 °C and maintained for 10 min. Spectral data were scanned over the range *m*/*z* 85–600. Relative peak areas were quantified as the ratio of the relative peak area divided by the peak area of the IS.^18^

Fatty acid extraction from wheat seedlings was performed using a previously described method.^19^ Briefly, freeze‐dried wheat seedling samples (10 mg) were placed in 15‐mL tubes containing 100 μL of pentadecanoic acid (1 mg mL^−1^ in chloroform; used as an IS) and 2.5 mL of a chloroform:methanol solution (2:1, v/v). For methylation, the concentrated samples were mixed with 0.3 mL 0.9 m boron trifluoride in methanol. Fatty acids were analyzed using a model 7890B GC (Agilent) flame ionization detector with a DB‐WAX column (122–7032, 30 m length, 0.25 mm diameter, 0.25 μm film thickness; Agilent). Nitrogen was used as the carrier gas at a flow rate of 1.00 mL min^−1^. The front inlet and detector temperatures were 250 °C. The oven temperature was maintained at 130 °C for 3 min and then increased at a rate of 20 °C min^−1^ to 230 °C. The final temperature was increased at a rate of 3 °C min^−1^ to 250 °C and maintained for 5 min.^19^ Both qualitative and quantitative analyses were performed by comparing the peak area ratios of the samples with those of FAME mixture as the standards.^19^

Chlorophyll extraction was performed as previously reported^20^; briefly, a freeze‐dried wheat seedling sample (10 mg) was mixed with 1 mL of methanol. The mixture was vortexed for 30 s and then sonicated for 30 min at 70 °C using a thermomixer (model 5355; Eppendorf AG, Hamburg, Germany). The sonicated samples were centrifuged at 800 × *g* and 4 °C for 10 min using a centrifugal concentrator (CC‐105; TOMY, Tokyo, Japan). A spectrophotometer (Optizen POP; Mecasys Co., Daejeon, Republic of Korea) was used to measure the absorbance of the supernatant at 666 and 653 nm. Total chlorophyll content was calculated using Wellburn's formula.^20^

### Extraction and analysis of secondary metabolites

The secondary lipophilic substances, such as policosanols, tocopherols and sterols, were extracted as previously described.^21^ Briefly, 10 mg of freeze‐dried wheat seedlings was extracted with 3 mL of 0.55 m ascorbic acid ethanol and 50 μL of 5α‐cholestane (10 μg mL^−1^) as an IS. Then, 30 μL of MSTFA and 30 μL of pyridine were added for derivatization, followed by incubation at 60 °C for 30 min in a thermomixer (model 5355; Eppendorf AG). For separation, 1‐μL aliquots were injected at a split ratio of 1:10 into a GC‐MS‐QP2010 Ultra system (Shimadzu, Kyoto, Japan) equipped with an Rtx‐5MS column (30 m × 0.25 mm, 0.25 μm inner diameter; Restek). Helium gas was delivered at a steady rate of 1 mL min^−1^. The inlet temperature was 290 °C. The oven temperature was initially 150 °C for 2 min, then increased to 320 °C at a rate of 15 °C min^−1^ and maintained for 10 min. The temperatures of the ion source and contact were 230 and 280 °C, respectively. Spectra were acquired over the range *m*/*z* 85–600 and ions were identified in selected ion‐monitoring mode for peak analysis. Chromatographic data were analyzed utilizing LabSolutions GCMS solution software, version 4.11 (Shimadzu). Accurate calibration curves for absolute quantification were established for each lipophilic standard at loadings ranging from 0.025 μg to 5.00 μg, with a fixed IS weight of 0.50 μg.^21^

The carotenoid extraction and determination procedure was slightly modified from previously published methods.^22^ Briefly, samples of freeze‐dried wheat seedlings (10 mg) were mixed with 3 mL of 0.55 m ascorbic acid in ethanol in a 15‐mL tube and vortexed. Water (1.5 mL), hexane (0.75 mL), toluene (0.75 mL) and 100 μL of 25 ppm trans‐*β*‐apo‐8‐carotenal (as an IS) were added. The concentrated extract was dissolved in 250 μL of a methanol:dichloromethane solution (1:1, v/v) and filtered using a 0.5‐μm syringe filter. Carotenoids were separated using an model 1100 HPLC system (Agilent) equipped with a diode‐array detector set to 450 nm, on a YMC carotenoid HPLC column (250 × 4.6 mm, 3 μm inner diameter) (YMC, Kyoto, Japan). A calibration curve was created by dividing the peak area of the IS by that of the standard compound for quantification.^22^

The extraction and estimation processes for flavonoid compounds were slightly modified from previously described methods.^23^ We placed 50 mg of wheat seedlings and 2 mL of 2.5 ppm galangin (IS) in methanol in a 5‐mL tube. The filtered samples (10 μL) were analyzed using a tandem MS and photodiode array detector connected to an ultra‐performance liquid chromatography (UPLC) system (ACQUITY UPLC I‐Class PLUS’ Waters, Milford, MA, USA). The UPLC was fitted with an ACQUITY UPLC CSH C18 1.7 μm column (150 × 2.1 mm, 1.7 μm column thickness; Waters). Quantification was performed using a calibration curve generated by dividing the IS peak area by the standard compound peak area.

### Statistical analysis

Each group comprised three biological replicates of wheat seedlings. Before multivariate analyses, the data were adjusted using unit variance scaling. Principal component analysis (PCA), partial least squares‐discriminant analysis (PLS‐DA) and projection to latent structure (PLS) were performed using SIMCA software, version 14.1; Umetrics, Umeå, Sweden). Pathway enrichment analysis based on the Kyoto Encyclopedia of Genes and Genomes (KEGG) (https://www.genome.jp/kegg) pathway database was conducted using MetaboAnalyst, version 6.0 (https://www.metaboanalyst.ca; accessed on 4 March 2024). Additionally, analysis of variance was performed using MetaboAnalyst, version 6.0 (accessed 4 March 2024) to determine significant differences [false discovery rate (FDR) < 0.05] among wheat seedlings grown under different PPFD conditions and growth periods.

## RESULTS AND DISCUSSION

### Metabolic profiling of wheat seedlings

Wheat seedlings are rich in secondary metabolites associated with antioxidant properties,^23^ and, during germination, environmental factors influence the concentrations of these metabolites.^2^ Primary metabolites, which have valuable nutritional properties, are directly involved in growth, development and reproduction, serving as components of secondary metabolites.^19^ Therefore, to understand the impact of environmental factors on the nutritional quality of wheat seedling metabolites, a comprehensive analysis of both primary and secondary metabolites is crucial. We conducted primary and secondary metabolic profiling of wheat seedlings cultivated under different PPFD conditions (200, 400 and 800 μmol m^−2^ s^−1^) and growth periods (5, 7, 9, 11, 13 and 15 days) and characterized a total of 74 metabolites, comprising 51 primary metabolites, including amino acids, fatty acids and organic acids, and 23 secondary metabolites, such as policosanols, sterols, flavonoids and carotenoids (see Supporting information, Tables S1–S18).

### Multivariate analyses of wheat seedlings cultivated under different photosynthetic photon flux densities and growth periods

Multivariate analyses are employed to establish the importance of key factors across numerous variables and unravel complex biochemical processes.^24^ To assess the impact of controlled light intensity and growth period on the metabolism of wheat seedlings, we performed multivariate analyses, including PCA, PLS‐DA, PLS modeling and pathway enrichment analysis. First, PCA was conducted to assess the overall correlation among wheat seedling samples grown under the various PPFD conditions and growth periods (see Supporting information, Fig. S1). This statistical technique reduces samples from a high‐dimensional space into a low‐dimensional space while preserving meaningful correlations.^25^ In our study, the PCA model revealed that the sum of the top two principal components (PCs) accounted for 44.8% of the total variance (29.7% for PC1 and 15.1% for PC2). PC1 clearly distinguished wheat seedlings cultivated under a PPFD of 200 μmol m^−2^ s^−1^ (PPFD 200) from those grown under PPFD of 400 μmol m^−2^ s^−1^ (PPFD 400) and 800 μmol m^−2^ s^−1^ (PPFD 800). With regard to the growth period, wheat seedlings grown for 5 days tended to be distinct from those grown for other growth periods, whereas wheat seedlings grown for more than 7 days did not show a clear separation. These results indicate that the effect of light intensity on the metabolism of wheat seedlings is stronger than that of growth period.

### Effects of photosynthetic photon flux densities on metabolite concentrations in wheat seedlings

**Figure 1:**
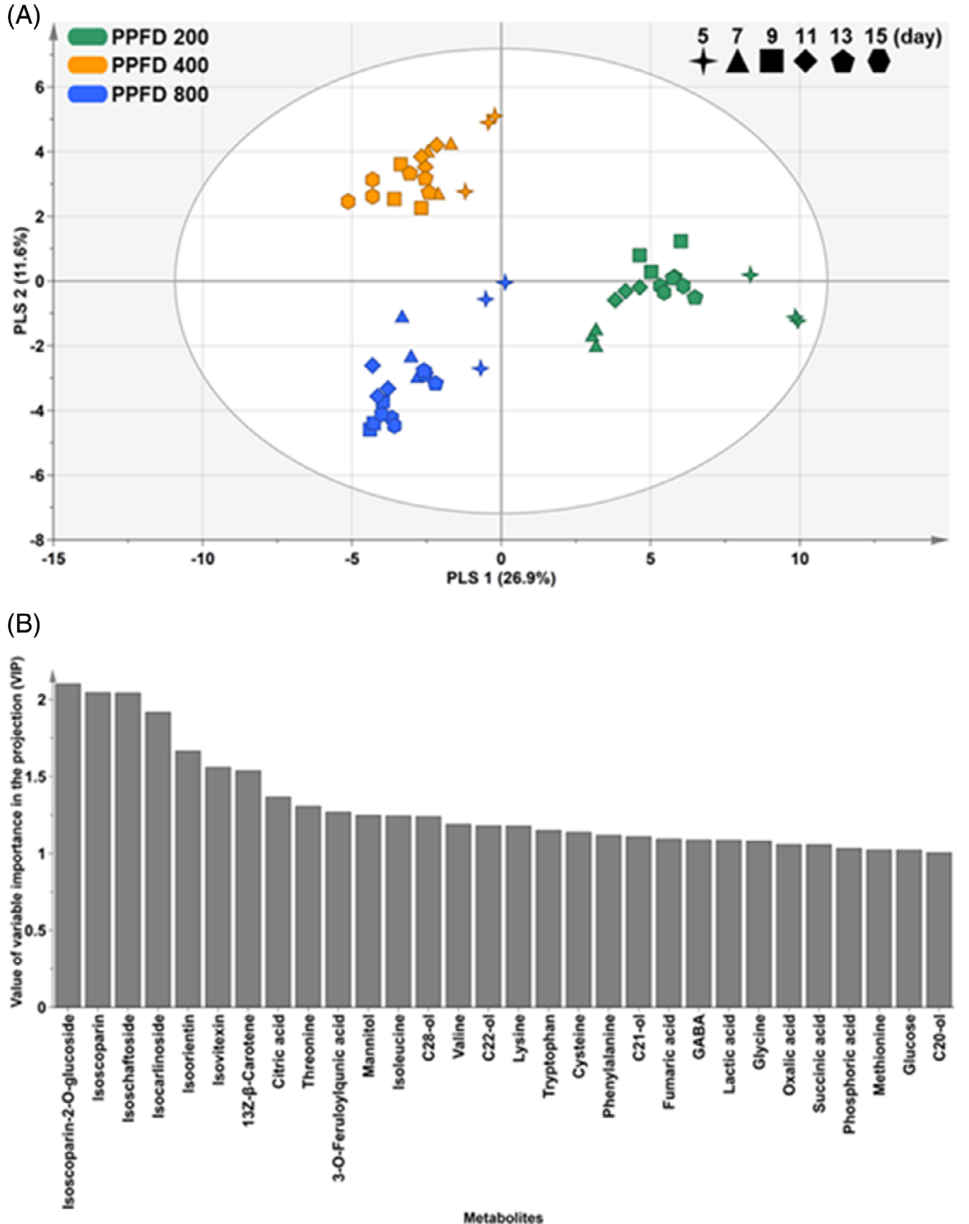
PLS‐DA score (A) and VIP (B) plots from 74 metabolites considering PPFD conditions. The VIP plot highlights metabolites with VIP > 1. PPFD, photosynthetic photon flux density; PLS‐DA, partial least squares‐discriminant analysis; VIP, variable importance in the projection.

Based on previous results, PLS‐DA was conducted to further examine the metabolic changes in wheat seedlings according to PPFD conditions (Fig. 1A). PLS‐DA is a statistical approach that rotates PCA to achieve maximum separation between groups under specified conditions.^26^ We used PPFD conditions as classification variables to distinguish models for PPFD 200, PPFD 400 and PPFD 800. The fit and predictive capability of the generated model were evaluated from their *R* ^2^ and *Q* ^2^ values, respectively. The generated PLS–DA model effectively represented the differences in samples based on light conditions, with high *R* ^2^ and *Q* ^2^ values (> 0.5). To identify the major metabolites contributing to this separation, a variable importance in the projection (VIP) plot of the respective PLS‐DA model was produced (Fig. 1B). In the VIP plot, metabolites with VIP > 1 are considered to substantially contribute to the separation of samples.^26^ In the present study, flavonoids such as isoscoparine‐2‐*O*‐glucoside, isoscoparine, isochaftoside, isocarinoside, isoorientin and isovitexin showed the highest VIP values. Previous studies characterized the phenolic metabolite profiles and antioxidant capacity of wheat seedling cultivars and growth periods.^23^ Wheat seedlings are a rich source of flavonoids and are beneficial to human health. To the best of our knowledge, no study has thus far reported metabolic changes in wheat seedlings influenced by PPFD conditions. Therefore, the present results confirm, for the first time, that changes in PPFD significantly impact flavonoid metabolism in wheat seedlings.

**Figure 2:**
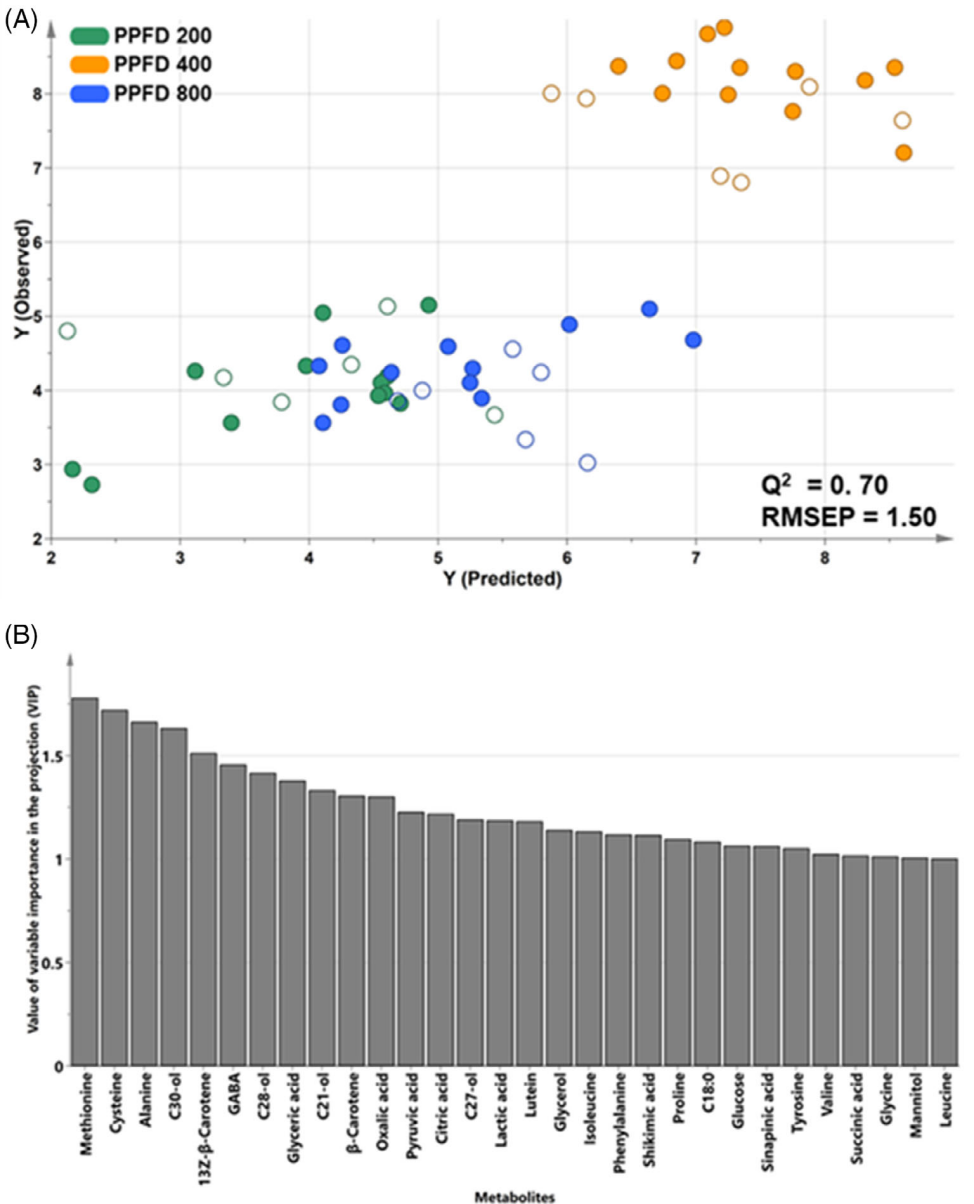
Score (A) and VIP (B) plots of a predictive PLS model built to predict total flavonoid content based on the metabolite profile of wheat seedlings. Wheat seedling samples were divided into 36 training sets (filled circles) and 18 test sets (open circles). The VIP plot highlights metabolites with VIP > 1. PLS, projection to latent structure; VIP, variable importance in the projection.

Plant secondary metabolites are derived from primary metabolites, thus a comprehensive understanding of the biosynthetic pathways of primary metabolites is necessary.^19^ However, interpretation of complex metabolic networks in wheat seedlings through primary and secondary metabolite profiling is still lacking. Therefore, a predictive PLS model based on the relationship between primary and secondary metabolites in wheat seedlings was fitted (Fig. 2). PLS involves extracting a new variable that maximizes the covariance between the linear combination of independent variables (*X*) and the dependent variable (*Y*).^21^ For model development, samples are divided into two groups: a training set, used to determine optimal parameters and create the model, and a test set, which is excluded from the regression analysis and used for validation.^19^, ^20^ The strength of the prediction is assessed using the cross‐validated correlation coefficient (*Q* ^2^) and root‐mean‐squared error of prediction (RMSEP). *Q* ^2^ > 0.5 indicates a good model,^27^ and a lower RMSEP value indicates a better fitting ability for the full range of data values for the dependent variable.^27^ Based on the significance of flavonoids under different PPFD conditions shown in the PLS‐DA model results, a PLS model was constructed using the total flavonoid content of each sample as the dependent variable (*Y*) and the 67 metabolites, excluding flavonoids, as independent variables (*X*). The wheat seedling samples were divided into 36 training and 18 test set samples (Fig. 2A). The model had a *Q* ^2^ of 0.7 and an RMSEP of 1.5, indicating that primary metabolite levels were accurately predicted based on the total flavonoid content (Fig. 2A). In the PLS score plot, wheat seedlings grown at PPFD 400 were predicted to contain the highest flavonoid levels compared to those grown under other conditions. Significant metabolites with VIP > 1 included methionine, cysteine, lutein, 13*Z*‐*ß*‐carotene, *ß*‐carotene, citric acid, phenylalanine, shikimic acid, succinic acid and tyrosine (Fig. 2B). These metabolites were classified into sulfur‐containing amino acids, carotenoids, intermediates of the TCA cycle and precursors of secondary metabolism. Light intensity influences photosynthetic performance, while sulfur‐containing amino acids such as methionine and cysteine affect the production of energy metabolites and secondary metabolites in response to light and redox signals.^28^, ^29^, ^30^ Accordingly, the PLS model supported the correlation between primary metabolism and the increased flavonoid content in wheat seedlings influenced by PPFD.

**Figure 3:**
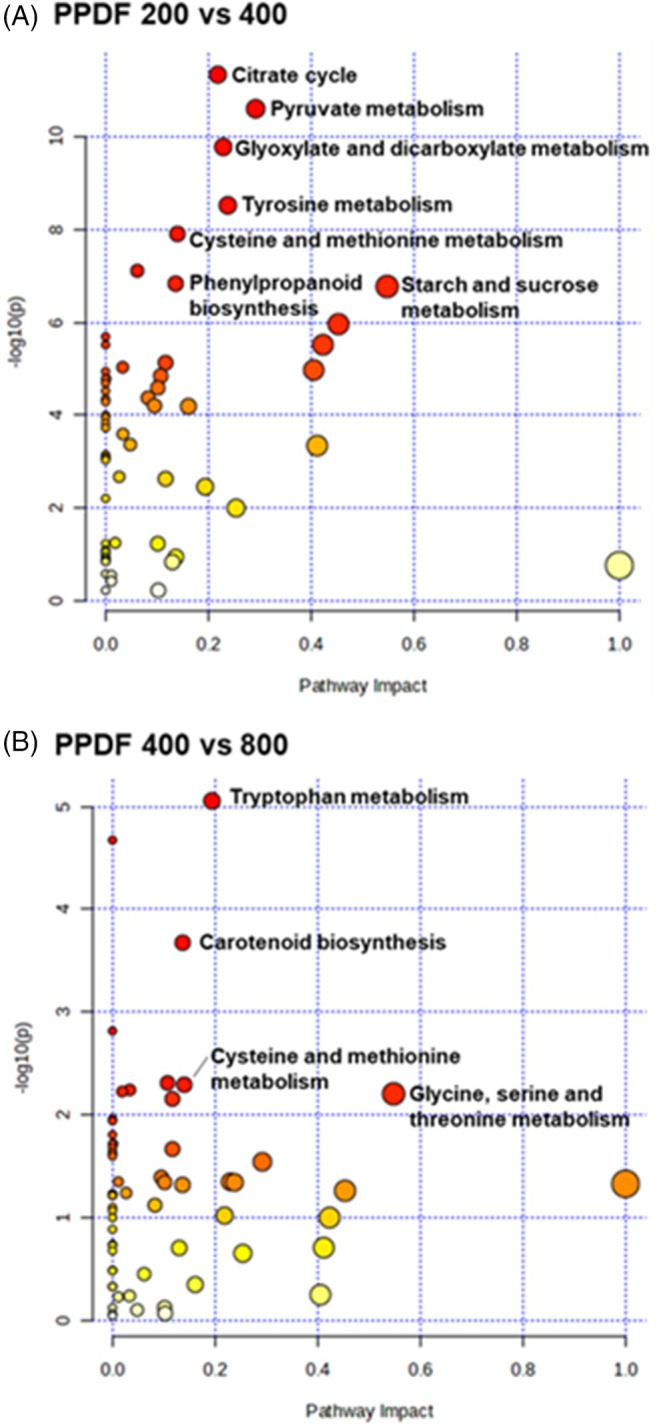
Overview of enrichment analysis by comparing wheat seedlings grown under different PPFD conditions: PPFD 200 *versus* PPFD 400 (A) and PPFD 400 *versus* PPFD 800 (B). The *x*‐axis represents the pathway impact value from pathway topological analysis and the *y*‐axis is the ‐log of the *P*‐value from pathway enrichment analysis. The circle size indicates the pathway impact score, and the circle color indicates adjusted *P*‐values, with red indicating higher values and white indicating lower values. PPFD 200, 200 μmol m^−2^ s^−1^; PPFD 400, 400 μmol m^−2^ s^−1^; PPFD 800, 800 μmol m^−2^ s^−1^.

To identify metabolic pathways in wheat seedlings that change in response to PPFD, pathway enrichment analysis was performed by comparing the treatment groups PPFD 200 with 400 and PPFD 400 with 800 (Fig. 3). Based on the metabolites identified in the KEGG database, six metabolic pathways were considered to be significant between wheat seedlings grown at PPFD 200 and 400 [−log(*p*) > 7.0 and impact score > 0.1]. These pathways included the TCA cycle, glyoxylate and dicarboxylate metabolism, pyruvate metabolism, starch and sucrose metabolism, tyrosine metabolism, phenylpropanoid biosynthesis, and cysteine and methionine metabolism (Fig. 3A). Additionally, between wheat seedlings grown at PPFD 400 and 800, the most affected pathways were tryptophan metabolism, carotenoid biosynthesis, cysteine and methionine metabolism, and glycine, serine and threonine metabolism (Fig. 3B).

**Figure 4:**
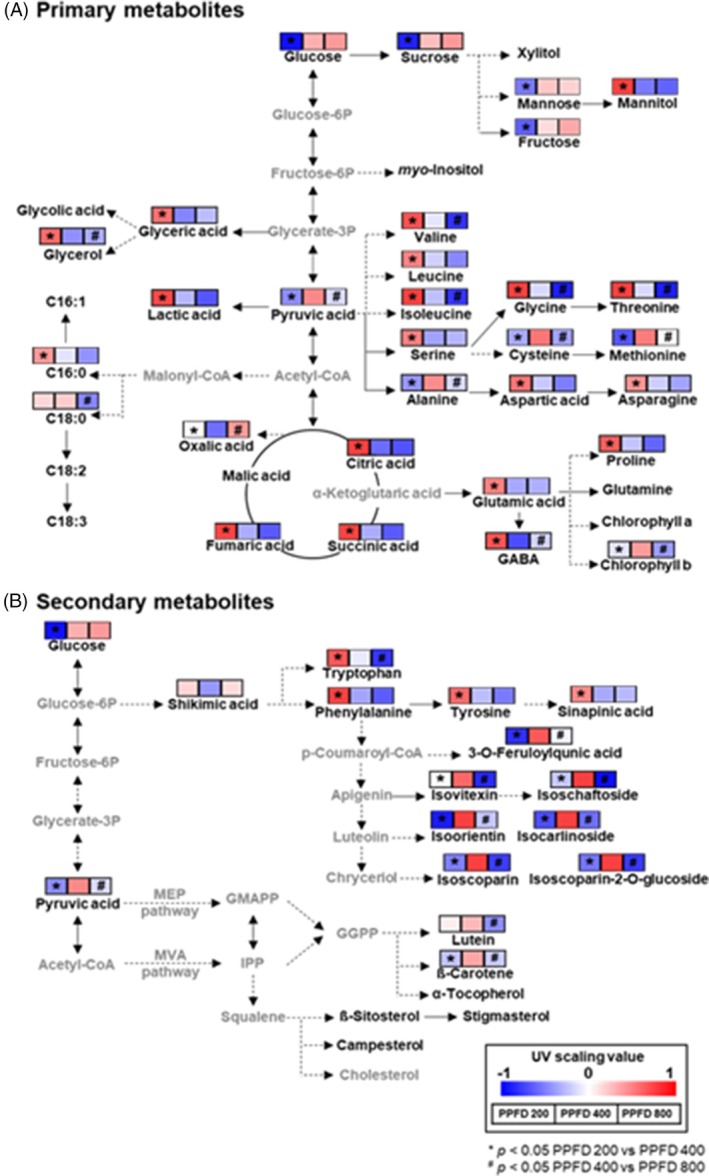
Primary (A) and secondary (B) metabolite biosynthesis pathways showing relative metabolite contents of wheat seedlings cultivated under different PPFD conditions. The metabolic pathway was adapted from the *Triticum aestivum* (bread wheat) KEGG pathway database. The colored squares (blue to red) indicate UV scaling values averaged for each metabolite. *P < 0.05 and #FDR < 0.05. KEGG, Kyoto Encyclopedia of Genes and Genomes; PCA, principal component analysis; PPFD, photosynthetic photon flux density.

Furthermore, to interpret metabolic changes in wheat seedlings in response to different PPFD conditions, a summary representation of enriched metabolic pathways using average UV scaling values is shown in Fig. 4. Intermediates of the TCA cycle and most amino acids showed higher contents under PPFD 200 conditions compared to other conditions (Fig. 4A). The balance of carbon and nitrogen metabolism is sensitive to plant growth and to defense and adaptation to environmental factors.^31^, ^32^ The TCA cycle is central to energy metabolism and, along with amino acid metabolism, is most active during the early growth period.^32^, ^33^ Wheat seedlings are typically grown at a PPFD of 100–200 μmol m^−2^ s^−1^, which is suitable for the vegetative stages of leaf growth.^32^, ^33^, ^34^ Our results showed that PPFD 200 conditions are generally the optimal condition for wheat seedling growth, consistent with the findings of previous studies. By contrast, sugar (glucose, fructose, mannose and sucrose), cysteine and methionine contents increased in PPFD 400 wheat seedlings (Fig. 4A). The contents of secondary metabolic precursors (shikimic acid, phenylalanine, tyrosine and tryptophan), carotenoids and flavonoids also increased under PPFD 400 conditions (Fig. 4B). Photosynthesis is an anabolic process in plants that produces glucose as an energy source, and the photosynthetic rate and sugar accumulation increase with PPFD.^35^ Sugars are the main source of secondary metabolites; accordingly, their accumulation affects the accumulation of secondary metabolites,^35^ which serve as plant defense compounds against stress caused by various factors.^30^ In addition, cysteine and methionine metabolism is known to regulate secondary metabolism depending on light intensity.^36^ The contents of sulfur‐containing amino acids cysteine and methionine increase with light intensity, and controlling PPFD enhances cysteine and methionine levels, thereby activating ethylene biosynthesis, which mediates stress response in various cells.^28^, ^36^, ^37^ In other words, the PPFD 400 condition induced active photosynthesis during the growth of wheat seedlings and enhanced sugar metabolism and defense mechanisms, resulting in an increased content of flavonoids, which are known antioxidant compounds. The content of carotenoids (lutein and ß‐carotene) was decreased (Fig. 4B), whereas that of oxalic acid was increased (Fig. 4A) in wheat seedlings grown under PPFD 800 conditions compared to those in seedlings grown under other conditions. PPFD 800 causes excessive excitation energy and photoinhibition compared to the typical growing conditions of wheat seedlings.^38^ Carotenoids are photosynthetic pigments for which the content increases with light intensity and decreases under excessive light conditions.^39^ Oxalic acid is an indicator of the oxidative stress caused by excessive light,^39^ and our results indicated that the PPFD 800 treatment resulted in excessive light exposure and inhibited wheat seedling growth. Taken together, our results confirmed that PPFD strongly affects the nutritional quality of wheat seedlings. The effects of the various PPFD conditions on secondary metabolites in wheat seedlings were clearly evident, and these changes were strongly correlated with primary metabolite changes. In conclusion, PPFD 400 conditions are optimal for increasing photosynthesis in wheat seedlings and producing flavonoids, which are beneficial to health.

### Effects of growth periods on metabolite contents of wheat seedlings

**Figure 5:**
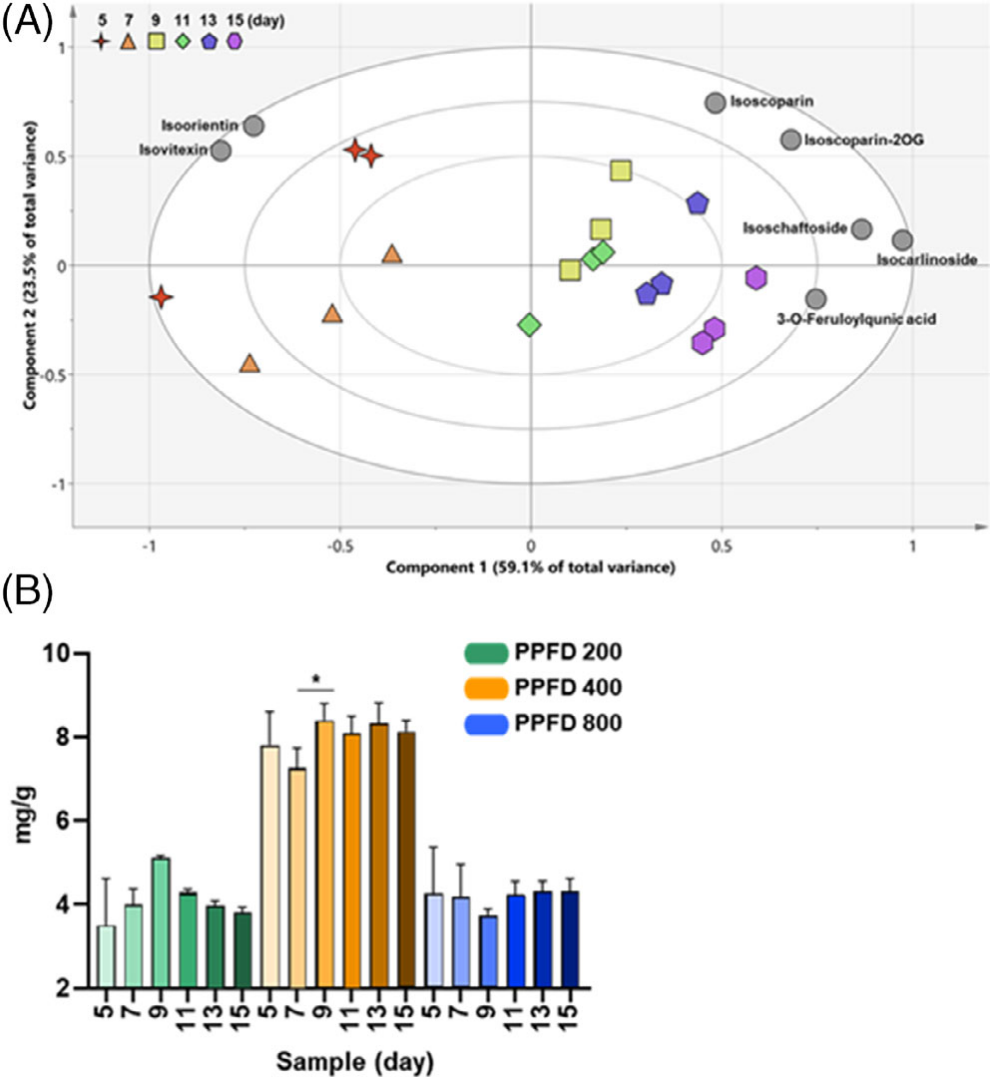
Biplot of PCs 1 and 2 from PCA generated from seven flavonoids in wheat seedlings grown under PPFD of 400 μmol m^−2^ s^−1^ at various growth periods (A). The bar graph represents the total flavonoid content (mean ± SD) of wheat seedlings grown under different PPFD conditions and growth periods (B). *P < 0.05. PCA, principal component analysis; PCs, principal components; PPFD, photosynthetic photon flux density.

Our results confirmed the excellent nutritional quality of flavonoids in wheat seedlings grown under PPFD 400 conditions. Accordingly, PCA was performed to determine the effect of growth periods under PPFD (Fig. 5). The relationships between samples and flavonoids are visualized in a biplot (Fig. 5A). In the PCA model, the top two PCs accounted for 82.6% of the total variance (59.1% for PC1 and 23.5% for PC2). Wheat seedlings grown for more than 9 days were separated from those grown for 5 and 7 days based on PC1. Most flavonoids showed higher content in wheat seedlings older than 9 days. Furthermore, as shown in the bar graph expressing total flavonoid content, flavonoid levels increased under the PPFD 400 condition compared to those under other conditions (Fig. 5B). Flavonoid content decreased in wheat seedlings grown under PPFD 400 for 7 days and then increased again after 9 days (*P* < 0.05) (Fig. 5B). Secondary metabolite content tends to increase as crops and plants mature; however, flavonoid content in seedlings can vary depending on factors including growth period and environmental conditions. In artificially cultivated sprouts, high levels of secondary metabolites are produced in a relatively short period of time, that is, approximately 6–8 days after germination.^40^ These changes can be attributed to flavonoids that are involved in various aspects of plant development, including seed germination, seedling development, flower color and fragrance.^41^ In our previous study, the flavonoid content of wheat seedlings (Saekeumkang cultivar) was highest on day 9, followed by those on day 7 > day 5 > day 12 > day 14,^17^ which is consistent with the results of the present study. Thus, the optimal cultivation conditions for wheat seedlings, as a source of flavonoids, were confirmed to be PPFD 400 and 9 days of growth.

## CONCLUSIONS

We performed a comprehensive profiling of the changes in primary and secondary metabolites in wheat seedlings under varying environmental factors such as PPFD conditions and growth periods. Multivariate analyses showed that the metabolism of wheat seedlings was more affected by PPFD than by the growth period. In the PLS‐DA model based on PPFD conditions, light intensity had a significant impact on flavonoid metabolism in wheat seedlings. In a prediction model that describes the relationship between primary and secondary metabolites in wheat seedlings, those grown under PPFD 400 were predicted to contain the highest flavonoid content. Enriched metabolic pathways in the response of wheat seedlings to PPFD included the TCA cycle, sugar metabolism, cysteine and methionine metabolism, carotenoid biosynthesis, and phenylpropanoid biosynthesis. Intermediates of the TCA cycle and most amino acids showed higher contents under PPFD 200 conditions than under other conditions. Sugars, pyruvic acid, cysteine, methionine, carotenoids and flavonoids increased in wheat seedlings cultivated under PPFD 400 conditions. Oxalic acid content increased in wheat seedlings grown under PPFD 800 conditions, which suggested that the PPFD 800 condition resulted in excessive light exposure to the wheat seedlings, thus affecting their growth. Compared with wheat seedlings grown under other conditions, the wheat seedlings grown at PPFD 400 for more than 9 days showed increased photosynthesis and high contents of health‐beneficial flavonoids. Based on these results, PPFD 200 can be applied in the early growth period to promote seedling development, and PPFD 400 is suitable for the later growth period when the production of functional ingredients such as flavonoids needs to be increased. Our results demonstrate the correlation between primary and secondary metabolites under PPFD conditions in wheat seedlings. Furthermore, this study supports the development of strategies for environmentally controlled cultivation to improve the nutritional and functional quality of wheat seedlings.

## CONFLICTS OF INTEREST

The authors declare no conflicts of interest.

## Supporting information


Data S1.
**Table S1**. Composition and content (ratio g^−1^) of hydrophilic compounds in wheat seedlings grown under a PPFD of 200 μmol m^−2^ s^−1^, analyzed by GC‐TOF‐MS.
**Table S2**. Composition and content (ratio g^−1^) of hydrophilic compounds in wheat seedlings grown under a PPFD of 400 μmol m^−2^ s^−1^, analyzed by GC‐TOF‐MS.
**Table S3**. Composition and content (ratio g^−1^) of hydrophilic compounds in wheat seedlings grown under a PPFD of 800 μmol m^−2^ s^−1^, analyzed by GC‐TOF‐MS.
**Table S4**. Composition and content (mg g^−1^) of fatty acid compounds in wheat seedlings grown under a PPFD of 200 μmol m^−2^ s^−1^, analyzed by GC‐FID.
**Table S5**. Composition and content (mg g^−1^) of fatty acid compounds in wheat seedlings grown under a PPFD of 400 μmol m^−2^ s^−1^, analyzed by GC‐FID.
**Table S6**. Composition and content (mg g^−1^) of fatty acid compounds in wheat seedlings grown under a PPFD of 800 μmol m^−2^ s^−1^, analyzed by GC‐FID.
**Table S7**. Composition and content (μg g^−1^) of chlorophyll compounds in wheat seedlings grown under a PPFD of 200 μmol m^−2^ s^−1^, analyzed by spectrophotometer.
**Table S8**. Composition and content (μg g^−1^) of chlorophyll compounds in wheat seedlings grown under a PPFD of 400 μmol m^−2^ s^−1^, analyzed by spectrophotometer.
**Table S9**. Composition and content (μg g^−1^) of chlorophyll compounds in wheat seedlings grown under a PPFD of 800 μmol m^−2^ s^−1^, analyzed by spectrophotometer.
**Table S10**. Composition and content (μg g^−1^) of lipophilic compounds in wheat seedlings grown under a PPFD of 200 μmol m^−2^ s^−1^, analyzed by GC‐MS.
**Table S11**. Composition and content (μg g^−1^) of lipophilic compounds in wheat seedlings grown under a PPFD of 400 μmol m^−2^ s^−1^, analyzed by GC‐MS.
**Table S12**. Composition and content (μg g^−1^) of lipophilic compounds in wheat seedlings grown under a PPFD of 800 μmol m^−2^ s^−1^, analyzed by GC‐MS.
**Table S13**. Composition and content (μg g^−1^) of carotenoid compounds in wheat seedling grown under a PPFD of 200 μmol m^−2^ s^−1^, analyzed by HPLC.
**Table S14**. Composition and content (μg g^−1^) of carotenoid compounds in wheat seedling grown under a PPFD of 400 μmol m^−2^ s^−1^, analyzed by HPLC.
**Table S15**. Composition and content (μg g^−1^) of carotenoid compounds in wheat seedling grown under a PPFD of 800 μmol m^−2^ s^−1^, analyzed by HPLC.
**Table S16**. Composition and content (μg g^−1^) of flavonoid compounds in wheat seedling grown under a PPFD of 200 μmol m^−2^ s^−1^, analyzed by UPLC‐MS/MS.
**Table S17**. Composition and content (μg g^−1^) of flavonoid compounds in wheat seedling grown under a PPFD of 400 μmol m^−2^ s^−1^, analyzed by UPLC‐MS/MS.
**Table S18**. Composition and content (μg g^−1^) of flavonoid compounds in wheat seedling grown under a PPFD of 800 μmol m^−2^ s^−1^, analyzed by UPLC‐MS/MS.
**Figure S1**. Score plot of principal component analysis (PCA) using 74 metabolites from wheat seedlings.

## Data Availability

The data that support the findings of this study are available from the corresponding author upon reasonable request.
