# Supplementary material for: Comparative analysis of primary and secondary metabolites in wheat seedlings ( Triticum aestivum L.) cultivated under varying photosynthetic photon flux densities and growth periods
Source: J Sci Food Agric. 2025 Jun 13;105(13):7260–9. doi: 10.1002/jsfa.14432 (PMC12439088; doi:10.1002/jsfa.14432)

Article

**Comparative analysis of primary and secondary metabolites in wheat seedlings (*Triticum aestivum* L.) cultivated under varying photosynthetic photon flux densities and growth periods**

Comparative Metabolic Analysis in Wheat Seedlings at Different Light Conditions

Ye Jin Kim ^a,#^, Yu–Mi Shin^a,#^, HanGyeol Lee^b^, So-Yeon Moon^b^, Tae Jin Kim^c^, Sang Un Park^d,#^, Woo Duck Seo^b,*^, Jae Kwang Kim^a,e,*^

^a^ Division of Life Sciences, Incheon National University, Yeonsu-gu, Incheon 22012, Republic of Korea

^b^ Division of Crop Foundation, National Institute of Crop Science, Rural Development Administration, Wanju, Jeonbuk 55365, Republic of Korea

^c^ Using Technology Development Department, Bio-resources Research Division, Nakdonggang National Institute of Biological Resources, Gyengsangbuk-do, 37242, Republic of Korea

^d^ Department of Crop Science, Chungnam National University, 99 Daehak-ro, Yuseong-gu, Daejeon34134, Republic of Korea

^e^ Convergence Research Center for Insect Vectors, College of Life Sciences and Bioengineering, Incheon National University, Incheon 22012, Republic of Korea

**List of supplementary table legends**

**Table S1.** Composition and content (ratio g^-1^) of hydrophilic compounds in wheat seedlings grown under a PPFD of 200 µmol m^-2^ s^-1^, analyzed by GC-TOF-MS.

**Table S2.** Composition and content (ratio g^-1^) of hydrophilic compounds in wheat seedlings grown under a PPFD of 400 µmol m^-2^ s^-1^, analyzed by GC-TOF-MS.

**Table S3.** Composition and content (ratio g^-1^) of hydrophilic compounds in wheat seedlings grown under a PPFD of 800 µmol m^-2^ s^-1^, analyzed by GC-TOF-MS.

**Table S4.** Composition and content (mg g^-1^) of fatty acid compounds in wheat seedlings grown under a PPFD of 200 µmol m^-2^ s^-1^, analyzed by GC-FID.

**Table S5.** Composition and content (mg g^-1^) of fatty acid compounds in wheat seedlings grown under a PPFD of 400 µmol m^-2^ s^-1^, analyzed by GC-FID.

**Table S6.** Composition and content (mg g^-1^) of fatty acid compounds in wheat seedlings grown under a PPFD of 800 µmol m^-2^ s^-1^, analyzed by GC-FID.

**Table S7.** Composition and content (µg g^-1^) of chlorophyll compounds in wheat seedlings grown under a PPFD of 200 µmol m^-2^ s^-1^, analyzed by spectrophotometer.

**Table S8.** Composition and content (µg g^-1^) of chlorophyll compounds in wheat seedlings grown under a PPFD of 400 µmol m^-2^ s^-1^, analyzed by spectrophotometer.

**Table S9.** Composition and content (µg g^-1^) of chlorophyll compounds in wheat seedlings grown under a PPFD of 800 µmol m^-2^ s^-1^, analyzed by spectrophotometer.

**Table S10**. Composition and content (µg g^-1^) of lipophilic compounds in wheat seedlings grown under a PPFD of 200 µmol m^-2^ s^-1^, analyzed by GC-MS.

**Table S11.** Composition and content (µg g^-1^) of lipophilic compounds in wheat seedlings grown under a PPFD of 400 µmol m^-2^ s^-1^, analyzed by GC-MS.

**Table S12.** Composition and content (µg g^-1^) of lipophilic compounds in wheat seedlings grown under a PPFD of 800 µmol m^-2^ s^-1^, analyzed by GC-MS.

**Table S13.** Composition and content (µg g^-1^) of carotenoid compounds in wheat seedling grown under a PPFD of 200 µmol m^-2^ s^-1^, analyzed by HPLC.

**Table S14.** Composition and content (µg g^-1^) of carotenoid compounds in wheat seedling grown under a PPFD of 400 µmol m^-2^ s^-1^, analyzed by HPLC.

**Table S15.** Composition and content (µg g^-1^) of carotenoid compounds in wheat seedling grown under a PPFD of 800 µmol m^-2^ s^-1^, analyzed by HPLC.

**Table S16.** Composition and content (µg g^-1^) of flavonoid compounds in wheat seedling grown under a PPFD of 200 µmol m^-2^ s^-1^, analyzed by UPLC-MS/MS.

**Table S17.** Composition and content (µg g^-1^) of flavonoid compounds in wheat seedling grown under a PPFD of 400 µmol m^-2^ s^-1^, analyzed by UPLC-MS/MS.

**Table S18.** Composition and content (µg g^-1^) of flavonoid compounds in wheat seedling grown under a PPFD of 800 µmol m^-2^ s^-1^, analyzed by UPLC-MS/MS.

**Figure S1.** Score plot of principal component analysis (PCA) using 74 metabolites from wheat seedlings.

| **Table S1.** Composition and content (ratio g^-1^) of hydrophilic compounds in wheat seedlings grown under a PPFD of 200 µmol m^-2^ s^-1^, analyzed by GC-TOF-MS. | | | | | | |
| --- | --- | --- | --- | --- | --- | --- |
| Compound | Contents (Average ± Standard Deviation, ratio g^-1^) | | | | | |
|  | 5 days | 7 days | 9 days | 11 days | 13 days | 15 days |
| Pyruvic acid | 0.28±0.02 | 0.58±0.02 | 0.36±0.01 | 0.05±0.00 | 0.42±0.01 | 0.44±0.03 |
| Lactic acid | 13.19±1.58 | 10.96±1.14 | 11.37±4.38 | 0.64±0.07 | 5.55±0.83 | 5.09±0.33 |
| Alanine | 469.59±19.81 | 544.05±19.99 | 546.64±11.18 | 49.16±1.28 | 495.16±9.48 | 516.46±21.39 |
| Oxalic acid | 53.32±2.46 | 53.41±5.83 | 47.97±1.03 | 5.13±0.13 | 59.83±4.96 | 53.08±3.21 |
| Glycolic acid | 0.26±0.01 | 0.24±0.01 | 0.21±0.01 | 0.02±0.00 | 0.24±0.01 | 0.30±0.02 |
| Valine | 281.90±14.54 | 190.97±2.07 | 231.48±3.6 | 20.84±0.43 | 265.40±6.29 | 259.92±11.03 |
| Serine | 16.89±0.74 | 18.44±0.20 | 19.09±1.2 | 2.23±0.09 | 24.09±0.86 | 24.08±3.22 |
| Glycerol | 216.55±4.32 | 160.84±1.26 | 181.79±3.64 | 13.68±0.11 | 134.81±3.78 | 130.46±2.76 |
| Ethanolamine | 46.37±2.49 | 35.70±1.64 | 39.36±0.57 | 3.25±0.02 | 31.92±0.70 | 28.79±1.43 |
| Phosphoric acid | 409.56±25.06 | 395.74±6.32 | 414.58±4.29 | 52.36±1.08 | 543.98±21.35 | 539.52±9.40 |
| Leucine | 247.74±9.63 | 144.92±3.91 | 163.82±3.01 | 14.33±0.16 | 146.87±3.43 | 146.97±6.20 |
| Isoleucine | 218.44±15.84 | 120.83±3.93 | 153.29±1.73 | 12.88±0.2 | 172.09±3.87 | 165.09±6.27 |
| Proline | 280.93±18.60 | 127.47±5.94 | 148.74±4.26 | 9.43±0.01 | 124.44±3.92 | 180.66±10.13 |
| Glycine | 118.37±6.44 | 75.44±2.19 | 79.80±1.70 | 8.64±0.11 | 73.77±1.71 | 65.28±1.89 |
| Succinic acid | 137.37±8.03 | 164.79±2.07 | 108.46±2.35 | 16.22±0.25 | 125.10±2.45 | 110.44±5.85 |
| Glyceric acid | 35.89±2.29 | 29.51±0.92 | 19.62±0.59 | 2.22±0.03 | 18.39±0.44 | 22.29±1.18 |
| Fumaric acid | 10.31±0.45 | 11.62±0.30 | 7.27±0.19 | 1.56±0.02 | 13.55±0.37 | 16.35±0.81 |
| Threonine | 18.37±1.26 | 14.69±0.08 | 17.29±0.14 | 1.54±0.04 | 18.93±0.44 | 20.08±0.38 |
| Beta-alanine | 12.63±1.16 | 5.55±0.09 | 6.16±0.21 | 0.34±0.00 | 4.32±0.14 | 5.29±0.22 |
| Malic acid | 73.54±5.49 | 144.84±2.56 | 125.25±1.79 | 14.95±0.18 | 165.67±3.91 | 212.96±3.29 |
| Aspartic acid | 99.47±7.36 | 67.79±4.06 | 100.47±1.5 | 8.07±0.26 | 103.15±7.09 | 95.69±4.40 |
| Methionine | 1.59±0.20 | 1.22±0.05 | 2.51±0.04 | 0.47±0.02 | 2.73±0.12 | 3.67±0.27 |
| Pyroglutamic acid | 33.80±3.19 | 18.61±0.58 | 28.54±1.00 | 2.20±0.04 | 26.40±0.95 | 26.95±1.68 |
| GABA^1^ | 72.99±1.55 | 57.92±0.96 | 62.64±1.62 | 6.22±0.13 | 62.76±2.38 | 68.58±1.36 |
| Glutamic acid | 5.88±0.74 | 6.09±0.08 | 7.11±0.27 | 0.44±0.02 | 5.76±0.24 | 7.29±0.28 |
| Phenylalanine | 83.13±7.88 | 46.21±0.76 | 60.83±0.99 | 4.82±0.10 | 67.54±1.85 | 74.03±3.46 |
| Asparagine | 148.91±7.10 | 180.52±2.53 | 201.00±3.45 | 22.02±0.39 | 261.88±5.72 | 236.87±8.41 |
| Xylitol | 2.86±0.21 | 2.71±0.06 | 3.13±0.16 | 0.31±0.01 | 3.25±0.14 | 2.79±0.09 |
| Glutamine | 24.24±2.06 | 9.57±0.19 | 16.63±1.06 | 0.70±0.02 | 14.11±0.87 | 15.63±0.62 |
| Shikimic acid | 11.05±0.89 | 20.00±0.58 | 10.58±0.25 | 1.27±0.01 | 9.99±0.25 | 18.34±1.35 |
| Citric acid | 51.7±4.02 | 47.34±0.54 | 63.39±1.18 | 6.62±0.14 | 78.03±1.34 | 63.03±2.53 |
| Quinic acid | 14.28±1.28 | 23.14±0.81 | 8.53±0.28 | 1.14±0.03 | 6.22±0.24 | 12.06±1.08 |
| Fructose | 284.42±12.51 | 329.04±4.35 | 237.82±5.15 | 32.92±0.65 | 183.44±1.50 | 353.68±9.63 |
| Mannose | 1.85±0.17 | 3.04±0.06 | 1.28±0.02 | 0.28±0.01 | 1.02±0.02 | 1.63±0.08 |
| Glucose | 66.01±3.50 | 157.64±1.78 | 102.23±1.16 | 18.17±0.66 | 70.00±2.13 | 109.63±3.45 |
| Mannitol | 6.55±0.42 | 5.49±0.30 | 25.75±0.66 | 1.67±0.07 | 18.59±0.52 | 24.44±0.66 |
| Lysine | 80.42±7.03 | 42.12±0.05 | 51.53±1.01 | 6.72±0.18 | 78.86±1.85 | 73.09±2.99 |
| Tyrosine | 118.43±13.04 | 53.29±0.34 | 72.96±1.36 | 5.81±0.13 | 74.81±1.43 | 80.89±4.72 |
| Inositol | 43.96±2.08 | 26.24±0.49 | 21.12±0.5 | 2.47±0.04 | 14.78±0.37 | 14.41±0.83 |
| Ferulic acid | 1.04±0.07 | 0.54±0.03 | 0.52±0.02 | 0.04±0.00 | 0.42±0.02 | 0.46±0.04 |
| Tryptophan | 30.03±3.15 | 16.61±0.37 | 21.35±0.66 | 1.64±0.03 | 27.85±0.72 | 27.65±1.54 |
| Sinapinic acid | 0.96±0.08 | 0.53±0.02 | 0.46±0.01 | 0.08±0.00 | 0.48±0.01 | 0.70±0.05 |
| Sucrose | 92.46±2.83 | 243.95±2.69 | 163.21±3.26 | 10.71±0.08 | 114.26±2.14 | 202.58±8.13 |
| Each value is the mean of three replications ± standard deviation (n=3). | | | |  |  |  |

^1^ Gamma-Aminobutyric acid

| **Table S2.** Composition and content (ratio g^-1^) of hydrophilic compounds in wheat seedlings grown under a PPFD of 400 µmol m^-2^ s^-1^, analyzed by GC-TOF-MS. | | | | | | |
| --- | --- | --- | --- | --- | --- | --- |
| Compound | Contents (Average ± Standard Deviation, ratio g^-1^) | | | | | |
|  | 5 days | 7 days | 9 days | 11 days | 13 days | 15 days |
| Pyruvic acid | 0.55±0.01 | 1.28±0.03 | 0.62±0 | 1.05±0.01 | 0.29±0.01 | 0.67±0.08 |
| Lactic acid | 5.18±1.84 | 4.52±0.78 | 4.91±0.09 | 5.44±0.35 | 4.91±0.29 | 4.91±0.03 |
| Alanine | 515.71±22.53 | 595.15±11.10 | 542.98±33.68 | 562.58±13.51 | 567.24±21.48 | 531.71±37.83 |
| Oxalic acid | 22.96±1.21 | 33.55±2.37 | 42.07±0.71 | 47.60±0.75 | 58.65±2.68 | 50.61±3.78 |
| Glycolic acid | 0.26±0.08 | 0.19±0.01 | 0.19±0.01 | 0.21±0.01 | 0.25±0.020 | 0.28±0.01 |
| Valine | 229.68±13.00 | 191.54±5.92 | 170.74±9.77 | 200.99±3.61 | 222.62±3.51 | 183.49±9.65 |
| Serine | 13.71±0.36 | 15.54±1.06 | 17.82±0.93 | 18.83±0.23 | 19.82±1.06 | 18.43±1.72 |
| Glycerol | 156.24±2.96 | 128.06±0.61 | 136.82±2.69 | 112.13±0.51 | 114.58±2.24 | 94.57±2.04 |
| Ethanolamine | 47.87±3.17 | 34.76±0.98 | 33.86±2.61 | 41.73±1.62 | 429.67±17.85 | 373.15±8.66 |
| Phosphoric acid | 409.24±22.27 | 481.24±8.30 | 485.96±10.67 | 472.79±6.06 | 41.96±1.69 | 39.83±1.28 |
| Leucine | 154.69±16.20 | 160.29±0.88 | 131.92±13.29 | 148.07±4.67 | 127.06±1.38 | 122.64±4.94 |
| Isoleucine | 136.65±18.26 | 115.70±2.36 | 97.06±9.5 | 107.83±3.75 | 115.09±1.60 | 90.26±5.50 |
| Proline | 132.95±23.77 | 122.98±4.01 | 92.17±11.96 | 107.20±6.15 | 94.10±2.73 | 85.33±8.86 |
| Glycine | 61.80±6.21 | 78.85±1.94 | 61.64±3.1 | 70.85±2.39 | 41.91±0.85 | 41.82±2.85 |
| Succinic acid | 51.22±2.91 | 135.63±1.42 | 102.99±1.95 | 109.17±2.04 | 44.51±1.09 | 71.83±3.47 |
| Glyceric acid | 18.49±0.77 | 22.20±0.26 | 21.65±0.58 | 17.37±1.10 | 13.21±0.59 | 17.21±1.02 |
| Fumaric acid | 3.28±0.12 | 10.33±0.09 | 6.75±0.11 | 10.03±0.05 | 3.81±0.07 | 9.18±0.53 |
| Threonine | 15.25±1.15 | 14.13±0.52 | 12.96±0.65 | 14.75±0.22 | 15.78±0.32 | 13.09±0.65 |
| Beta-alanine | 10.81±0.86 | 4.49±0.07 | 3.69±0.11 | 3.51±0.06 | 5.26±0.08 | 3.23±0.13 |
| Malic acid | 64.46±3.62 | 92.15±1.97 | 123.68±2.61 | 133.25±1.49 | 156.77±2.19 | 167.14±4.60 |
| Aspartic acid | 112.32±6.33 | 59.60±3.4 | 51.31±1.94 | 54.29±0.75 | 73.59±2.18 | 43.83±3.40 |
| Methionine | 7.64±0.59 | 4.06±0.19 | 2.93±0.04 | 5.78±0.04 | 5.11±0.07 | 4.65±0.61 |
| Pyroglutamic acid | 36.71±0.50 | 18.01±0.41 | 18.15±0.39 | 19.02±0.73 | 20.90±1.38 | 17.61±0.41 |
| GABA^1^ | 59.66±1.98 | 55.32±0.45 | 54.88±1.91 | 55.09±0.65 | 58.40±0.66 | 52.75±1.30 |
| Glutamic acid | 5.76±0.84 | 3.14±0.18 | 4.57±0.24 | 3.50±0.01 | 6.24±0.10 | 4.52±0.34 |
| Phenylalanine | 56.79±4.37 | 44.83±0.60 | 34.53±0.67 | 40.29±0.66 | 46.47±0.77 | 37.71±1.84 |
| Asparagine | 230.80±3.62 | 189.23±6.89 | 149.72±2.09 | 166.87±1.80 | 154.80±4.72 | 134.29±8.52 |
| Xylitol | 2.35±0.09 | 2.96±0.10 | 3.26±0.06 | 3.79±0.06 | 3.88±0.32 | 3.41±0.19 |
| Glutamine | 36.86±0.83 | 7.91±0.49 | 6.37±0.12 | 7.54±0.28 | 11.73±0.23 | 6.31±0.45 |
| Shikimic acid | 5.44±0.31 | 9.66±0.09 | 13.43±0.22 | 11.22±0.11 | 10.69±0.42 | 16.19±0.95 |
| Citric acid | 36.62±2.16 | 26.57±0.27 | 28.56±0.76 | 32.63±0.37 | 35.90±0.32 | 29.00±2.03 |
| Quinic acid | 5.33±0.35 | 11.14±0.12 | 20.29±0.51 | 13.31±0.23 | 10.25±0.23 | 16.18±1.25 |
| Fructose | 170.18±9.65 | 409.88±8.34 | 425.88±7.23 | 427.14±4.14 | 294.46±19.82 | 554.22±14.20 |
| Mannose | 0.82±0.04 | 4.03±0.06 | 3.84±0.05 | 4.17±0.05 | 1.15±0.09 | 4.40±0.21 |
| Glucose | 124.31±3.67 | 260.26±4.26 | 249.59±7.76 | 263.10±8.91 | 134.87±10.55 | 263.93±5.52 |
| Mannitol | 1.32±0.13 | 1.85±0.51 | 2.24±0.04 | 4.10±0.20 | 2.80±0.43 | 2.82±0.11 |
| Lysine | 47.86±2.26 | 55.27±0.88 | 45.67±0.71 | 64.87±1.03 | 56.16±1.88 | 48.96±3.51 |
| Tyrosine | 82.58±3.95 | 59.49±1.40 | 42.39±0.74 | 49.17±0.79 | 51.89±0.98 | 42.95±2.63 |
| Inositol | 19.47±0.59 | 26.94±0.47 | 25.46±0.42 | 22.99±0.34 | 15.53±0.28 | 19.23±1.08 |
| Ferulic acid | 0.81±0.03 | 0.65±0.01 | 0.6±0.01 | 0.59±0.00 | 0.61±0.05 | 0.61±0.06 |
| Tryptophan | 19.23±1.22 | 15.83±0.61 | 15.35±0.3 | 18.70±0.34 | 22.87±0.55 | 18.33±1.25 |
| Sinapinic acid | 0.23±0.01 | 0.50±0.02 | 0.81±0.01 | 0.43±0.00 | 0.34±0.01 | 0.46±0.03 |
| Sucrose | 156.60±3.08 | 203.94±4.45 | 246.67±3.98 | 211.38±1.34 | 264.44±7.73 | 285.69±3.84 |
| Each value is the mean of three replications ± standard deviation (n=3).  ^1^ Gamma-Aminobutyric acid | | | |  |  |  |

| **Table S3.** Composition and content (ratio g^-1^) of hydrophilic compounds in wheat seedlings grown under a PPFD of 800 µmol m^-2^ s^-1^, analyzed by GC-TOF-MS. | | | | | | |
| --- | --- | --- | --- | --- | --- | --- |
| Compound | Contents (Average ± Standard Deviation, ratio g^-1^) | | | | | |
|  | 5 days | 7 days | 9 days | 11 days | 13 days | 15 days |
| Pyruvic acid | 0.33±0.02 | 0.87±0.08 | 0.32±0.06 | 0.94±0.08 | 0.23±0.00 | 0.38±0.06 |
| Lactic acid | 3.45±0.25 | 4.07±1.27 | 4.37±0.39 | 3.73±0.26 | 3.49±0.19 | 3.84±0.30 |
| Alanine | 508.02±36.28 | 575.15±52.65 | 492.28±33.79 | 483.22±36.86 | 526.71±15.75 | 539.58±45.03 |
| Oxalic acid | 36.17±8.56 | 44.01±7.16 | 45.74±9.90 | 63.38±1.95 | 79.77±5.06 | 100.83±3.87 |
| Glycolic acid | 0.27±0.04 | 0.24±0.01 | 0.19±0.00 | 0.26±0.00 | 0.25±0.04 | 0.26±0.01 |
| Valine | 215.64±11.11 | 171.05±9.27 | 145.89±10.32 | 151.00±16.58 | 183.97±2.57 | 166.09±13.22 |
| Serine | 17.28±1.28 | 15.28±1.81 | 22.33±0.61 | 20.64±1.30 | 17.05±0.56 | 13.10±0.33 |
| Glycerol | 165.42±0.55 | 131.47±2.58 | 131.87±3.89 | 111.59±1.74 | 123.36±2.33 | 96.40±2.94 |
| Ethanolamine | 45.74±5.81 | 30.39±2.31 | 406.94±21.97 | 413.81±17.33 | 398.38±10.35 | 372.78±12.71 |
| Phosphoric acid | 415.36±3.13 | 460.35±18.42 | 31.58±0.40 | 39.89±1.18 | 44.31±3.57 | 36.59±1.70 |
| Leucine | 152.29±9.82 | 144.64±13.37 | 111.48±7.06 | 125.62±12.22 | 135.37±6.65 | 125.47±8.97 |
| Isoleucine | 115.35±12.61 | 95.44±10.63 | 68.95±5.37 | 75.02±8.69 | 96.01±3.64 | 88.18±6.68 |
| Proline | 109.73±16.29 | 103.62±16.68 | 61.80±5.99 | 86.19±15.96 | 88.92±4.26 | 73.49±6.86 |
| Glycine | 70.81±6.72 | 56.44±2.65 | 47.38±2.55 | 56.52±2.02 | 37.09±1.52 | 29.06±1.34 |
| Succinic acid | 39.58±1.02 | 110.55±2.81 | 79.83±2.81 | 92.66±1.89 | 57.2±1.05 | 77.03±2.61 |
| Glyceric acid | 18.26±0.32 | 21.66±0.66 | 20.09±0.75 | 17.47±0.13 | 16.25±0.23 | 22.96±0.82 |
| Fumaric acid | 2.40±0.05 | 7.70±0.20 | 5.28±0.20 | 7.76±0.19 | 4.26±0.09 | 8.33±0.28 |
| Threonine | 13.87±1.34 | 12.49±1.24 | 10.53±0.69 | 11.92±0.44 | 13.95±0.49 | 11.70±0.85 |
| Beta-alanine | 10.77±1.01 | 3.62±0.12 | 3.00±0.16 | 2.72±0.04 | 4.19±0.06 | 2.82±0.12 |
| Malic acid | 52.17±1.36 | 106.53±2.36 | 99.14±3.09 | 123.25±0.51 | 159.20±1.43 | 206.53±6.87 |
| Aspartic acid | 118.93±5.58 | 51.96±0.62 | 41.88±1.10 | 43.13±0.86 | 57.27±1.44 | 34.87±1.30 |
| Methionine | 6.61±0.49 | 3.45±0.15 | 2.11±0.16 | 4.33±0.20 | 3.58±0.16 | 3.02±0.13 |
| Pyroglutamic acid | 53.57±2.05 | 19.81±1.34 | 17.98±0.67 | 21.71±0.78 | 23.25±0.19 | 19.52±0.63 |
| GABA^1^ | 62.48±1.33 | 56.61±0.73 | 52.87±2.18 | 57.29±0.47 | 62.79±1.39 | 60.41±1.43 |
| Glutamic acid | 7.76±1.08 | 3.37±0.21 | 3.77±0.17 | 3.27±0.22 | 4.63±0.20 | 5.16±0.19 |
| Phenylalanine | 50.08±3.89 | 43.50±1.24 | 28.29±1.41 | 34.78±0.91 | 43.35±1.29 | 36.2±1.00 |
| Asparagine | 285.38±4.31 | 166.18±5.09 | 148.18±3.33 | 144.35±2.93 | 130.59±1.09 | 101.31±4.10 |
| Xylitol | 2.19±0.06 | 3.03±0.06 | 3.32±0.09 | 3.69±0.11 | 3.56±0.11 | 3.02±0.12 |
| Glutamine | 75.68±0.93 | 12.61±0.42 | 11.69±0.45 | 16.30±0.65 | 13.14±0.65 | 6.80±0.39 |
| Shikimic acid | 5.79±0.21 | 11.72±0.25 | 12.83±0.46 | 15.10±0.34 | 15.89±0.41 | 20.33±0.57 |
| Citric acid | 30.37±1.20 | 26.52±0.88 | 20.83±0.94 | 29.67±0.91 | 37.56±0.55 | 34.74±0.87 |
| Quinic acid | 5.87±0.26 | 13.40±0.28 | 20.91±0.59 | 19.71±0.41 | 16.50±0.46 | 22.56±0.32 |
| Fructose | 196.91±5.95 | 450.78±8.43 | 448.12±10.35 | 441.61±7.55 | 376.53±7.50 | 549.32±29.98 |
| Mannose | 0.85±0.03 | 4.32±0.14 | 4.29±0.17 | 4.01±0.10 | 1.52±0.03 | 2.91±0.23 |
| Glucose | 134.94±3.57 | 285.67±7.00 | 277.93±8.46 | 257.12±12.11 | 158.37±3.62 | 230.29±13.38 |
| Mannitol | 0.70±0.04 | 1.66±0.46 | 1.63±0.05 | 2.33±0.02 | 2.29±0.09 | 2.28±0.13 |
| Lysine | 45.41±1.73 | 47.59±1.43 | 36.96±1.58 | 48.07±0.55 | 43.84±0.76 | 32.70±1.51 |
| Tyrosine | 72.99±4.54 | 57.39±1.78 | 35.65±1.67 | 40.48±1.36 | 47.58±1.03 | 42.04±1.33 |
| Inositol | 18.57±0.48 | 26.83±0.73 | 23.17±0.74 | 21.89±0.47 | 17.42±0.21 | 19.61±0.63 |
| Ferulic acid | 0.99±0.11 | 0.79±0.06 | 0.65±0.04 | 0.65±0.01 | 0.63±0.02 | 0.55±0.03 |
| Tryptophan | 15.17±1.89 | 15.63±0.37 | 12.98±0.57 | 13.27±0.36 | 16.28±0.36 | 15.44±0.61 |
| Sinapinic acid | 0.19±0.01 | 0.48±0.02 | 0.69±0.03 | 0.45±0.02 | 0.44±0.01 | 0.59±0.02 |
| Sucrose | 169.44±4.12 | 239.14±5.16 | 259.06±9.03 | 210.45±3.71 | 257.55±8.11 | 301.21±8.81 |
| Each value is the mean of three replications ± standard deviation (n=3).  ^1^ Gamma-Aminobutyric acid | | | |  |  |  |

| **Table S4.** Composition and content (mg g^-1^) of fatty acid compounds in wheat seedlings grown under a PPFD of 200 µmol m^-2^ s^-1^, analyzed by GC-FID. | | | | | | |
| --- | --- | --- | --- | --- | --- | --- |
| Compound | Contents (Average ± Standard Deviation, mg g^-1^) | | | | | |
|  | 5 days | 7 days | 9 days | 11 days | 13 days | 15 days |
| C16:0 (Palmitic acid) | 5.45±0.20 | 4.23±0.12 | 4.07±0.38 | 3.87±0.13 | 3.71±0.25 | 3.80±0.18 |
| C16:1n7 (Palmitoleic acid) | 0.33±0.03 | 0.38±0.06 | 0.37±0.02 | 0.36±0.02 | 0.36±0.05 | 0.35±0.03 |
| C18:0 (Stearic acid) | 0.80±0.05 | 0.71±0.05 | 0.80±0.07 | 0.71±0.07 | 0.69±0.06 | 0.75±0.03 |
| C18:2n6 (Linoleic acid) | 7.79±0.31 | 6.30±0.26 | 6.02±0.59 | 5.21±0.13 | 4.88±0.21 | 5.04±0.21 |
| C18:3n3 (α-Linolenic acid) | 52.94±1.87 | 46.36±0.89 | 49.08±4.60 | 46.78±0.88 | 44.60±1.90 | 46.61±2.00 |
| Each value is the mean of three replications ± standard deviation (n=3). | | | | | | |

| **Table S5.** Composition and content (mg g^-1^) of fatty acid compounds in wheat seedlings grown under a PPFD of 400  µmol m^-2^ s^-1^, analyzed by GC-FID. | | | | | | |
| --- | --- | --- | --- | --- | --- | --- |
| Compound | Contents (Average ± Standard Deviation, mg g^-1^) | | | | | |
|  | 5 days | 7 days | 9 days | 11 days | 13 days | 15 days |
| C16:0 (Palmitic acid) | 4.26±0.09 | 3.87±0.03 | 3.85±0.13 | 3.99±0.19 | 3.74±0.12 | 3.71±0.13 |
| C16:1n7 (Palmitoleic acid) | 0.34±0.01 | 0.33±0.02 | 0.37±0.03 | 0.38±0.05 | 0.37±0.00 | 0.38±0.04 |
| C18:0 (Stearic acid) | 0.78±0.05 | 0.69±0.03 | 0.77±0.06 | 0.79±0.09 | 0.73±0.07 | 0.73±0.08 |
| C18:2n6 (Linoleic acid) | 6.63±0.09 | 5.36±0.03 | 5.38±0.12 | 5.36±0.32 | 5.11±0.04 | 4.87±0.08 |
| C18:3n3 (α-Linolenic acid) | 50.54±1.02 | 48.83±0.28 | 32.25±27.42 | 31.17±26.38 | 44.97±0.26 | 43.98±0.96 |
| Each value is the mean of three replications ± standard deviation (n=3). | | | | | | |

| **Table S6.** Composition and content (mg g^-1^) of fatty acid compounds in wheat seedlings grown under a PPFD of 800 µmol m^-2^ s^-1^, analyzed by GC-FID. | | | | | | |
| --- | --- | --- | --- | --- | --- | --- |
| Compound | Contents (Average ± Standard Deviation, mg g^-1^) | | | | | |
|  | 5 days | 7 days | 9 days | 11 days | 13 days | 15 days |
| C16:0 (Palmitic acid) | 4.10±0.11 | 3.76±0.05 | 3.78±0.11 | 3.62±0.20 | 3.83±0.13 | 3.49±0.11 |
| C16:1n7 (Palmitoleic acid) | 0.32±0.02 | 0.32±0.03 | 0.36±0.01 | 0.33±0.01 | 0.40±0.03 | 0.37±0.06 |
| C18:0 (Stearic acid) | 0.75±0.08 | 0.65±0.03 | 0.72±0.07 | 0.65±0.04 | 0.74±0.06 | 0.67±0.06 |
| C18:2n6 (Linoleic acid) | 6.10±0.14 | 5.13±0.12 | 5.10±0.05 | 4.64±0.30 | 4.98±0.13 | 4.54±0.15 |
| C18:3n3 (α-Linolenic acid) | 46.65±1.20 | 45.95±0.94 | 44.76±0.20 | 45.06±2.55 | 45.16±1.03 | 41.97±1.35 |
| Each value is the mean of three replications ± standard deviation (n=3). | | | | | | |

| **Table S7.** Composition and content (µg g^-1^) of chlorophyll compounds in wheat seedlings grown under a PPFD of 200 µmol m^-2^ s^-1^, analyzed by spectrophotometer. | | | | | | |
| --- | --- | --- | --- | --- | --- | --- |
| Compound | Contents (Average ± Standard Deviation, µg g^-1^) | | | | | |
|  | 5 days | 7 days | 9 days | 11 days | 13 days | 15 days |
| Chlorophyll a | 6.43±0.15 | 8.29±0.05 | 8.34±0.35 | 9.10±0.40 | 9.03±0.22 | 9.78±0.34 |
| Chlorophyll b | 2.75±0.10 | 3.37±0.09 | 3.27±0.13 | 3.66±0.11 | 3.61±0.09 | 4.35±0.16 |
| Each value is the mean of three replications ± standard deviation (n=3). | | | | | | |

| **Table S8.** Composition and content (µg g^-1^) of chlorophyll compounds in wheat seedlings grown under a PPFD of 400 µmol m^-2^ s^-1^, analyzed by spectrophotometer. | | | | | | |
| --- | --- | --- | --- | --- | --- | --- |
| Compound | Contents (Average ± Standard Deviation, µg g^-1^) | | | | | |
|  | 5 days | 7 days | 9 days | 11 days | 13 days | 15 days |
| Chlorophyll a | 7.93±0.25 | 9.11±0.18 | 9.07±0.26 | 8.59±0.13 | 9.68±0.50 | 9.89±0.59 |
| Chlorophyll b | 3.25±0.18 | 3.89±0.15 | 4.10±0.08 | 3.75±0.05 | 3.79±0.45 | 4.00±0.66 |
| Each value is the mean of three replications ± standard deviation (n=3). | | | | | | |

| **Table S9.** Composition and content (µg g^-1^) of chlorophyll compounds in wheat seedlings grown under a PPFD of 800 µmol m^-2^ s^-1^, analyzed by spectrophotometer. | | | | | | |
| --- | --- | --- | --- | --- | --- | --- |
| Compound | Contents (Average ± Standard Deviation, µg g^-1^) | | | | | |
|  | 5 days | 7 days | 9 days | 11 days | 13 days | 15 days |
| Chlorophyll a | 7.82±0.15 | 9.69±0.55 | 9.06±0.12 | 10.03±0.47 | 9.48±0.86 | 9.06±0.18 |
| Chlorophyll b | 2.76±0.21 | 3.59±0.27 | 3.54±0.05 | 3.53±0.51 | 3.26±0.16 | 3.55±0.07 |
| Each value is the mean of three replications ± standard deviation (n=3). | | | | | | |

| **Table S10.** Composition and content (µg g^-1^) of lipophilic compounds in wheat seedlings grown under a PPFD of 200 µmol m^-2^ s^-1^, analyzed by GC-MS. | | | | | | |
| --- | --- | --- | --- | --- | --- | --- |
| Compound | Contents (Average ± Standard Deviation, µg g^-1^) | | | | | |
|  | 5 days | 7 days | 9 days | 11 days | 13 days | 15 days |
| C20-ol (Arachidyl alcohol) | 18.23±0.42 | 19.15±1.49 | 19.83±1.75 | 19.39±0.76 | 19.21±1.65 | 16.38±0.68 |
| C21-ol (heneicosanol) | 8.41±0.91 | 9.31±0.65 | 9.94±1.12 | 10.35±0.41 | 7.06±0.71 | 6.51±0.64 |
| C22-ol (Docosanol) | 49.71±6.77 | 63.19±4.95 | 70.94±3.07 | 68.49±3.1 | 63.74±5.14 | 43.75±4.99 |
| C24-ol (Tetracosanol) | 38.31±2.44 | 36.96±1.13 | 39.00±2.33 | 39.57±1.71 | 40.50±1.46 | 39.10±1.73 |
| C26-ol (Hexacosanol) | 240.89±23.12 | 224.21±28.44 | 220.41±33.97 | 218.46±11.09 | 206.45±3.09 | 183.13±4.94 |
| C27-ol (Heptacosanol) | 54.05±3.13 | 59.71±2.22 | 59.10±4.13 | 56.12±7.91 | 57.67±6.08 | 54.53±1.59 |
| C28-ol (Octacosanol) | 16753.53±1607.17 | 15030.72±664.31 | 15559.82±1658.33 | 15047.80±678.60 | 15631.34±1451.39 | 13532.99±973.46 |
| α-Tocopherol | 74.03±5.69 | 95.79±5.40 | 100.76±1.54 | 98.43±5.06 | 82.53±7.37 | 93.67±17.82 |
| Campesterol | 369.75±36.25 | 284.06±16.49 | 343.48±22.39 | 352.75±31.60 | 308.94±16.26 | 247.03±23.39 |
| C30-ol (Triacontanol) | 563.06±66.98 | 689.72±32.96 | 645.46±69.20 | 654.84±12.44 | 605.36±25.25 | 508.43±66.68 |
| Stigmasterol | 118.82±12.43 | 124.75±7.56 | 165.79±4.97 | 186.12±5.93 | 209.85±21.20 | 162.28±2.55 |
| β-Sitosterol | 372.22±25.26 | 285.55±25.74 | 359.72±18.34 | 369.27±14.36 | 346.31±21.90 | 259.63±4.66 |
| Each value is the mean of three replications ± standard deviation (n=3). | | | | | | |

| **Table S11.** Composition and content (µg g^-1^) of lipophilic compounds in wheat seedlings grown under a PPFD of 400 µmol m^-2^ s^-1^, analyzed by GC-MS. | | | | | | |
| --- | --- | --- | --- | --- | --- | --- |
| Compound | Contents (Average ± Standard Deviation, µg g^-1^) | | | | | |
|  | 5 days | 7 days | 9 days | 11 days | 13 days | 15 days |
| C20-ol (Arachidyl alcohol) | 15.31±0.93 | 15.54±0.70 | 16.57±1.19 | 16.55±0.52 | 17.88±1.03 | 18.06±0.99 |
| C21-ol (heneicosanol) | 5.72±0.39 | 5.23±0.30 | 6.32±1.54 | 6.07±0.54 | 6.58±0.73 | 6.92±0.60 |
| C22-ol (Docosanol) | 25.97±2.32 | 29.76±1.89 | 38.91±8.50 | 40.74±2.92 | 38.94±4.14 | 64.57±10.65 |
| C24-ol (Tetracosanol) | 37.21±2.73 | 37.09±1.77 | 39.40±1.79 | 41.21±1.91 | 41.84±4.43 | 45.92±5.60 |
| C26-ol (Hexacosanol) | 280.64±39.13 | 263.14±34.96 | 296.07±5.54 | 294.88±23.72 | 263.46±15.66 | 281.46±39.01 |
| C27-ol (Heptacosanol) | 52.99±6.55 | 50.44±3.96 | 58.27±3.87 | 52.00±2.56 | 56.37±2.87 | 63.61±9.70 |
| C28-ol (Octacosanol) | 15590.86±1887.37 | 15721.63±1286.08 | 17731.26±974.58 | 14459.74±623.21 | 15466.94±1073.92 | 16953.65±2011.92 |
| α-Tocopherol | 91.33±4.77 | 69.98±12.42 | 103.46±4.44 | 84.40±3.76 | 89.87±7.27 | 116.00±19.95 |
| Campesterol | 398.05±61.45 | 337.40±21.76 | 361.02±28.47 | 315.36±4.09 | 337.85±34.95 | 296.51±51.34 |
| C30-ol (Triacontanol) | 547.91±104.33 | 491.36±179.61 | 504.49±68.63 | 428.92±90.86 | 415.23±44.93 | 587.51±67.23 |
| Stigmasterol | 134.44±15.91 | 121.06±19.40 | 166.73±4.52 | 145.37±8.47 | 208.94±36.66 | 201.64±31.38 |
| β-Sitosterol | 391.48±56.44 | 299.18±25.73 | 362.25±30.46 | 336.83±12.30 | 377.98±14.45 | 359.01±59.65 |
| Each value is the mean of three replications ± standard deviation (n=3). | | | | | | |

| **Table S12.** Composition and content (µg g^-1^) of lipophilic compounds in wheat seedlings grown under a PPFD of 800 µmol m^-2^ s^-1^, analyzed by GC-MS. | | | | | | |
| --- | --- | --- | --- | --- | --- | --- |
| Compound | Contents (Average ± Standard Deviation, µg g^-1^) | | | | | |
|  | 5 days | 7 days | 9 days | 11 days | 13 days | 15 days |
| C20-ol (Arachidyl alcohol) | 15.75±0.50 | 16.15±0.87 | 16.61±0.51 | 16.13±0.61 | 15.97±0.58 | 16.88±1.00 |
| C21-ol (heneicosanol) | 5.94±0.11 | 5.66±0.73 | 6.42±0.55 | 5.86±0.73 | 6.26±0.85 | 6.75±0.25 |
| C22-ol (Docosanol) | 27.32±0.55 | 28.11±3.78 | 36.71±5.60 | 33.18±4.07 | 31.30±4.21 | 34.51±1.21 |
| C24-ol (Tetracosanol) | 35.46±1.08 | 40.59±2.17 | 42.53±1.01 | 42.94±0.71 | 40.82±2.93 | 40.31±2.59 |
| C26-ol (Hexacosanol) | 284.48±29.85 | 375.81±13.83 | 304.74±32.22 | 310.38±5.90 | 198.18±18.88 | 255.02±26.92 |
| C27-ol (Heptacosanol) | 53.99±4.48 | 62.25±2.61 | 63.92±4.01 | 58.59±4.97 | 51.70±12.94 | 59.85±0.37 |
| C28-ol (Octacosanol) | 17356.60±1432.06 | 18852.34±856.02 | 22039.04±1715.30 | 17940.57±297.75 | 16660.06±1346.35 | 17979.32±943.01 |
| α-Tocopherol | 107.55±20.85 | 84.06±15.01 | 91.62±4.71 | 91.77±10.53 | 75.48±13.78 | 101.23±7.75 |
| Campesterol | 395.35±22.09 | 301.40±17.03 | 341.67±56.34 | 336.98±3.79 | 304.21±52.08 | 274.98±55.88 |
| C30-ol (Triacontanol) | 479.57±24.88 | 577.51±55.78 | 642.68±50.62 | 575.21±14.78 | 473.18±59.27 | 464.35±73.24 |
| Stigmasterol | 143.60±8.72 | 125.85±19.63 | 149.05±15.85 | 166.39±22.98 | 183.34±38.57 | 175.02±12.38 |
| β-Sitosterol | 373.68±49.18 | 299.31±11.67 | 357.57±36.75 | 334.83±7.16 | 357.03±60.66 | 316.08±20.81 |
| Each value is the mean of three replications ± standard deviation (n=3). | | | | | | |

| **Table S13.** Composition and content (µg g^-1^) of carotenoid compounds in wheat seedling grown under a PPFD of 200 µmol m^-2^ s^-1^, analyzed by HPLC. | | | | | | |
| --- | --- | --- | --- | --- | --- | --- |
| Compound | Contents (Average ± Standard Deviation, µg g^-1^) | | | | | |
|  | 5 days | 7 days | 9 days | 11 days | 13 days | 15 days |
| lutein | 885.9±22.98 | 998.05±35.58 | 1240.48±157.56 | 1139.12±52.19 | 982.61±24.43 | 1105.32±41.63 |
| trans-β-Apo-8'-carotenal | 326.77±9.97 | 329.42±13.18 | 326.69±7.43 | 329.22±8.57 | 329.19±7.93 | 324.29±9.26 |
| 13Z-β-carotene | 57.82±3.00 | 91.26±3.26 | 100.98±22.63 | 90.33±5.96 | 55.68±4.07 | 70.71±6.15 |
| β-carotene | 235.48±6.13 | 373.12±14.03 | 550.59±47.48 | 562.80±13.72 | 465.94±19.61 | 551.47±20.83 |
| 9Z-β-carotene | 35.34±0.37 | 54.85±3.30 | 69.71±7.54 | 72.32±3.75 | 62.46±3.13 | 69.37±3.01 |
| Each value is the mean of three replications ± standard deviation (n=3). | | | |  |  |  |

| **Table S14.** Composition and content (µg g^-1^) of carotenoid compounds in wheat seedling grown under a PPFD of 400 µmol m^-2^ s^-1^, analyzed by HPLC. | | | | | | |
| --- | --- | --- | --- | --- | --- | --- |
| Compound | Contents (Average ± Standard Deviation, µg g^-1^) | | | | | |
|  | 5 days | 7 days | 9 days | 11 days | 13 days | 15 days |
| lutein | 1073.33±14.55 | 1056.32±79.12 | 1060.79±49.41 | 1021.67±9.92 | 1176.77±106.34 | 1128.56±69.87 |
| trans-β-Apo-8'-carotenal | 311.37±7.68 | 316.04±10.42 | 324.15±4.19 | 310.18±5.14 | 329.25±9.44 | 319.62±12.03 |
| 13Z-β-carotene | 63.85±4.95 | 51.29±1.49 | 72.36±5.60 | 72.35±9.91 | 74.85±2.93 | 64.69±7.27 |
| β-carotene | 516.06±12.46 | 501.41±45.13 | 501.67±18.92 | 474.46±20.18 | 551.54±55.61 | 540.03±29.20 |
| 9Z-β-carotene | 65.06±1.93 | 62.36±5.23 | 61.04±1.24 | 53.54±2.39 | 67.30±8.40 | 65.09±3.06 |
| Each value is the mean of three replications ± standard deviation (n=3). | | | |  |  |  |

| **Table S15.** Composition and content (µg g^-1^) of carotenoid compounds in wheat seedling grown under a PPFD of 800 µmol m^-2^ s^-1^, analyzed by HPLC. | | | | | | |
| --- | --- | --- | --- | --- | --- | --- |
| Compound | Contents (Average ± Standard Deviation, µg g^-1^) | | | | | |
|  | 5 days | 7 days | 9 days | 11 days | 13 days | 15 days |
| lutein | 979.65±14.14 | 917.76±34.35 | 973.65±17.34 | 1034.12±57.91 | 1025.92±44.00 | 1088.42±26.04 |
| trans-β-Apo-8'-carotenal | 324.34±10.36 | 321.93±10.6 | 325.90±16.56 | 327.97±8.77 | 314.81±8.63 | 304.98±12.51 |
| 13Z-β-carotene | 94.47±4.58 | 92.67±7.00 | 87.07±4.37 | 93.00±2.33 | 95.05±3.43 | 107.28±7.00 |
| β-carotene | 442.95±17.52 | 432.07±8.97 | 446.65±11.27 | 493.94±26.96 | 451.89±18.27 | 486.74±0.63 |
| 9Z-β-carotene | 57.73±3.14 | 57.36±1.69 | 58.64±2.70 | 59.57±3.77 | 57.93±3.21 | 59.53±0.64 |
| Each value is the mean of three replications ± standard deviation (n=3). | | | |  |  |  |

| **Table S16.** Composition and content (µg g^-1^) of flavonoid compounds in wheat seedling grown under a PPFD of 200 µmol m^-2^ s^-1^, analyzed by UPLC-MS/MS. | | | | | | |
| --- | --- | --- | --- | --- | --- | --- |
| Compound | Contents (Average ± Standard Deviation, µg g^-1^) | | | | | |
|  | 5 days | 7 days | 9 days | 11 days | 13 days | 15 days |
| 3-O-Feruloylqunic acid | 14.99±8.03 | 14.66±0.99 | 8.45±0.34 | 4.77±0.30 | 4.40±0.32 | 11.63±1.16 |
| Isovitexin | 141.89±46.43 | 151.33±5.53 | 154.89±9.12 | 114.54±8.42 | 100.99±8.16 | 76.87±2.08 |
| Isoorientin | 1066.47±62.24 | 1108.86±38.25 | 1200.19±30.44 | 978.62±33.49 | 892.25±32.65 | 747.51±24.48 |
| Isoscoparin | 663.63±62.93 | 580.07±39.31 | 764.16±27.89 | 624.70±29.26 | 598.60±24.34 | 584.98±13.02 |
| Isochaftoside | 36.73±38.19 | 50.82±7.85 | 66.53±6.23 | 51.82±3.28 | 47.32±2.34 | 48.39±2.51 |
| Isocarlinoside | 893.85±539.03 | 1105.06±144.30 | 1630.20±29.89 | 1350.38±51.31 | 1169.71±33.45 | 1213.37±50.88 |
| Isoscoparin-2-o-glucoside | 670.94±415.78 | 982.88±155.60 | 1285.54±23.38 | 1164.75±28.31 | 1152.40±53.05 | 1125.59±35.59 |
| Each value is the mean of three replications ± standard deviation (n=3). | | | |  |  |  |

| **Table S17.** Composition and content (µg g^-1^) of flavonoid compounds in wheat seedling grown under a PPFD of 400 µmol m^-2^ s^-1^, analyzed by UPLC-MS/MS. | | | | | | |
| --- | --- | --- | --- | --- | --- | --- |
| Compound | Contents (Average ± Standard Deviation, µg g^-1^) | | | | | |
|  | 5 days | 7 days | 9 days | 11 days | 13 days | 15 days |
| 3-O-Feruloylqunic acid | 14.99±8.03 | 14.66±0.99 | 8.45±0.34 | 4.77±0.30 | 4.40±0.32 | 11.63±1.16 |
| Isovitexin | 141.89±46.43 | 151.33±5.53 | 154.89±9.12 | 114.54±8.42 | 100.99±8.16 | 76.87±2.08 |
| Isoorientin | 1066.47±62.24 | 1108.86±38.25 | 1200.19±30.44 | 978.62±33.49 | 892.25±32.65 | 747.51±24.48 |
| Isoscoparin | 663.63±62.93 | 580.07±39.31 | 764.16±27.89 | 624.70±29.26 | 598.60±24.34 | 584.98±13.02 |
| Isochaftoside | 36.73±38.19 | 50.82±7.85 | 66.53±6.23 | 51.82±3.28 | 47.32±2.34 | 48.39±2.51 |
| Isocarlinoside | 893.85±539.03 | 1105.06±144.30 | 1630.20±29.89 | 1350.38±51.31 | 1169.71±33.45 | 1213.37±50.88 |
| Isoscoparin-2-o-glucoside | 670.94±415.78 | 982.88±155.60 | 1285.54±23.38 | 1164.75±28.31 | 1152.40±53.05 | 1125.59±35.59 |
| Each value is the mean of three replications ± standard deviation (n=3). | | | |  |  |  |

| **Table S18.** Composition and content (µg g^-1^) of flavonoid compounds in wheat seedling grown under a PPFD of 800 µmol m^-2^ s^-1^, analyzed by UPLC-MS/MS. | | | | | | |
| --- | --- | --- | --- | --- | --- | --- |
| Compound | Contents (Average ± Standard Deviation, µg g^-1^) | | | | | |
|  | 5 days | 7 days | 9 days | 11 days | 13 days | 15 days |
| 3-O-Feruloylqunic acid | 14.99±8.03 | 14.66±0.99 | 8.45±0.34 | 4.77±0.30 | 4.40±0.32 | 11.63±1.16 |
| Isovitexin | 141.89±46.43 | 151.33±5.53 | 154.89±9.12 | 114.54±8.42 | 100.99±8.16 | 76.87±2.08 |
| Isoorientin | 1066.47±62.24 | 1108.86±38.25 | 1200.19±30.44 | 978.62±33.49 | 892.25±32.65 | 747.51±24.48 |
| Isoscoparin | 663.63±62.93 | 580.07±39.31 | 764.16±27.89 | 624.70±29.26 | 598.60±24.34 | 584.98±13.02 |
| Isochaftoside | 36.73±38.19 | 50.82±7.85 | 66.53±6.23 | 51.82±3.28 | 47.32±2.34 | 48.39±2.51 |
| Isocarlinoside | 893.85±539.03 | 1105.06±144.30 | 1630.20±29.89 | 1350.38±51.31 | 1169.71±33.45 | 1213.37±50.88 |
| Isoscoparin-2-o-glucoside | 670.94±415.78 | 982.88±155.60 | 1285.54±23.38 | 1164.75±28.31 | 1152.40±53.05 | 1125.59±35.59 |
| Each value is the mean of three replications ± standard deviation (n=3). | | | |  |  |  |

**Figure S1.** Score plot of principal component analysis (PCA) using 74 metabolites from wheat seedlings. PPFD 200, 200 µmol m^-2^ s^-1^; PPFD 400, 400 µmol m^-2^ s^-1^; PPFD 800, 800 µmol m^-2^ s^-1^


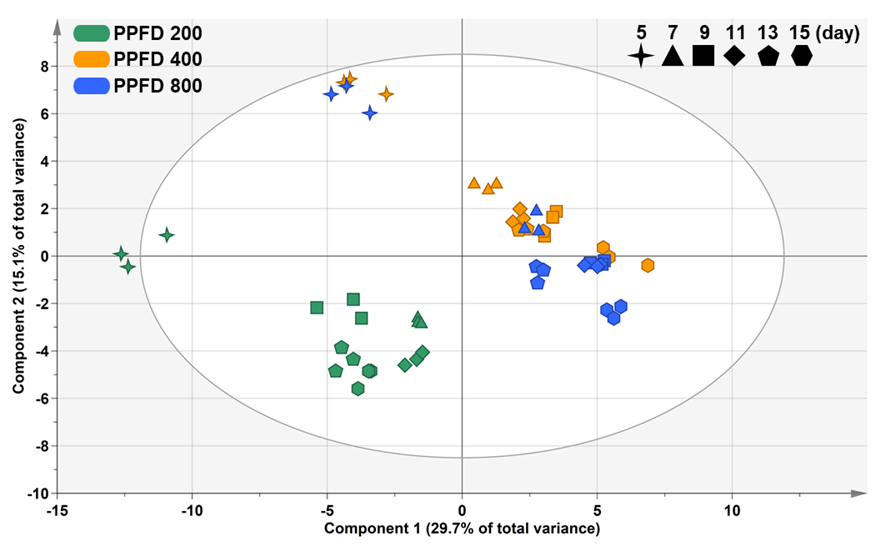

Supplement: Supplementary file 1 — Data S1. Table S1. Composition and content (ratio g−1) of hydrophilic compounds in wheat seedlings grown under a PPFD of 200 μmol m−2 s−1, analyzed by GC‐TOF‐MS. Table S2. Composition and content (ratio g−1) of hydrophilic compounds in wheat seedlings grown under a PPFD of 400 μmol m−2 s−1, analyzed by GC‐TOF‐MS. Table S3. Composition and content (ratio g−1) of hydrophilic compounds in wheat seedlings grown under a PPFD of 800 μmol m−2 s−1, analyzed by GC‐TOF‐MS. Table S4. Composition and content (mg g−1) of fatty acid compounds in wheat seedlings grown under a PPFD of 200 μmol m−2 s−1, analyzed by GC‐FID. Table S5. Composition and content (mg g−1) of fatty acid compounds in wheat seedlings grown under a PPFD of 400 μmol m−2 s−1, analyzed by GC‐FID. Table S6. Composition and content (mg g−1) of fatty acid compounds in wheat seedlings grown under a PPFD of 800 μmol m−2 s−1, analyzed by GC‐FID. Table S7. Composition and content (μg g−1) of chlorophyll compounds in wheat seedlings grown under a PPFD of 200 μmol m−2 s−1, analyzed by spectrophotometer. Table S8. Composition and content (μg g−1) of chlorophyll compounds in wheat seedlings grown under a PPFD of 400 μmol m−2 s−1, analyzed by spectrophotometer. Table S9. Composition and content (μg g−1) of chlorophyll compounds in wheat seedlings grown under a PPFD of 800 μmol m−2 s−1, analyzed by spectrophotometer. Table S10. Composition and content (μg g−1) of lipophilic compounds in wheat seedlings grown under a PPFD of 200 μmol m−2 s−1, analyzed by GC‐MS. Table S11. Composition and content (μg g−1) of lipophilic compounds in wheat seedlings grown under a PPFD of 400 μmol m−2 s−1, analyzed by GC‐MS. Table S12. Composition and content (μg g−1) of lipophilic compounds in wheat seedlings grown under a PPFD of 800 μmol m−2 s−1, analyzed by GC‐MS. Table S13. Composition and content (μg g−1) of carotenoid compounds in wheat seedling grown under a PPFD of 200 μmol m−2 s−1, analyzed by HPLC. Table S14. Composition and content (μg [file JSFA-105-7260-s001.docx]
